# Donor age and long-term culture do not negatively influence the stem potential of limbal fibroblast-like stem cells

**DOI:** 10.1186/s13287-016-0342-z

**Published:** 2016-06-13

**Authors:** Laura Tomasello, Rosa Musso, Giovanni Cillino, Maria Pitrone, Giuseppe Pizzolanti, Antonina Coppola, Walter Arancio, Gianluca Di Cara, Ida Pucci-Minafra, Salvatore Cillino, Carla Giordano

**Affiliations:** Laboratory of Regenerative Medicine, Section of Endocrinology, Diabetology and Metabolism, Di.Bi.M.I.S., University of Palermo, Piazza delle Cliniche 2, 90127 Palermo, Italy; Centro di Oncobiologia Sperimentale (COBS), Palermo, Italy; Department of Ophthalmology, University of Palermo, Palermo, Italy; ATeN (Advanced Technologies Network Center), University of Palermo, Palermo, Italy

**Keywords:** Regenerative medicine, Limbal stem cells, Fibroblast-like stem cells, Proteomic profile, Adult stem cell pluripotency

## Abstract

**Background:**

In regenerative medicine the maintenance of stem cell properties is of crucial importance. Ageing is considered a cause of reduced stemness capability. The limbus is a stem niche of easy access and harbors two stem cell populations: epithelial stem cells and fibroblast-like stem cells. Our aim was to investigate whether donor age and/or long-term culture have any influence on stem cell marker expression and the profiles in the fibroblast-like stem cell population.

**Methods:**

Fibroblast-like stem cells were isolated and digested from 25 limbus samples of normal human corneo-scleral rings and long-term cultures were obtained. SSEA4 expression and sphere-forming capability were evaluated; cytofluorimetric assay was performed to detect the immunophenotypes HLA-DR, CD45, and CD34 and the principle stem cell markers ABCG2, OCT3/4, and NANOG. Molecular expression of the principal mesenchymal stem cell genes was investigated by real-time PCR. Two-dimensional gel electrophoresis and mass spectrometric sequencing were performed and a stable proteomic profile was identified. The proteins detected were explored by gene ontology and STRING analysis. The data were reported as means ± SD, compared by Student’s unpaired *t* test and considering *p* < 0.05 as statistically significant.

**Results:**

The isolated cells did not display any hematopoietic surface marker (CD34 and CD45) and HLA-DR and they maintained these features in long-term culture. The expression of the stemness genes and the multilineage differentiation under in-vitro culture conditions proved to be well maintained. Proteomic analysis revealed a fibroblast-like stem cell profile of 164 proteins with higher expression levels. Eighty of these showed stable expression levels and were involved in maintenance of “the stem gene profile”; 84 were differentially expressed and were involved in structural activity.

**Conclusions:**

The fibroblast-like limbal stem cells confirmed that they are a robust source of adult stem cells and that they have good plasticity, good proliferative capability, and long-term maintenance of stem cell properties, independently of donor age and long-term culture conditions. Our findings confirm that limbal fibroblast-like stem cells are highly promising for application in regenerative medicine and that in-vitro culture steps do not influence their stem cell properties. Moreover, the proteomic data enrich our knowledge of fibroblast-like stem cells.

**Electronic supplementary material:**

The online version of this article (doi:10.1186/s13287-016-0342-z) contains supplementary material, which is available to authorized users.

## Background

The limbus, located at the junction of the cornea and conjunctiva of the ocular surface, is characterized by stromal invaginations (the palisades of Vogt). These structures give anatomical and functional properties protecting stem cells from insults and allowing constant renewal of the corneal epithelium [1–8].

Two stem cell populations have been described in the limbus: limbal epithelial stem cells (LESCs) and limbal stromal stem cells. Their similar and different features have not been defined unequivocally [9–12]. Polisetty et al. [13] proposed a role for limbal mesenchymal stromal cells (MSCs) in maintaining support of the stem cell niche. More recent data have validated the hypothesis of “niche stromal cells” based on the capacity of limbal stromal cells to be an efficient feeder layer for ex-vivo limbal epithelial cell expansion [8, 14, 15]. However, given the complexity of the limbal niche structure and its cellular components, which cellular type has the main role in normal tissue maintenance remains to be seen [16–18].

We previously identified a subpopulation of limbal stem cells, which we referred to as fibroblast-like limbal stem cells (f-LSCs) [19]. We indicated a core set of attributes that uniquely characterize f-LSCs and that classify them as mesenchymal stem cells. In support of this, f-LSCs expressed stem cell surface antigens SSEA4 (stage-specific embryonic antigen-4), TRA 1–60, and TRA 1–81 and several nuclear transcription factors, such as OCT4 (octamer-binding transcription factor 4), NANOG (Homeobox protein NANOG), and SOX2 (SRY (sex determining region Y)-box 2), involved in self-renewal and maintenance of pluripotency of both embryonic and adult stem cells [20, 21]. The f-LSCs were positive for the limbal stem cell marker ABCG2 (ATP-binding cassette sub-family G member 2) and were negative for the LESC marker ΔNp63 (a splice variant of p63) [22–26]. More recently it has been hypothesized that age negatively affects stem cell number and potential, also referring to the LESC population [27, 28].

The current study was undertaken to explore whether ex-vivo expansion of f-LSCs could modify their stem molecular features. Our study aimed to increase knowledge in the field of limbal mesenchymal stem cell research regarding three facets: firstly, we aimed to discover whether long-term culture affects the gene expression and proteomic profile of f-LSCs; secondly, we proposed a preserved “proteomic stem cell pattern” in ageing and long-term culture conditions; and finally, we evaluated the maintenance of multilineage differentiation capability in vitro.

## Methods

### Establishment of limbal cell cultures

Human tissues were used in accordance with the Declaration of Helsinki and informed written consent was given by each patient. The study was approved by the Ethical Committee of the AOUP, University of Palermo (No. 09/2009).

Normal human corneo-scleral rings from donors aged between 24 and 74 years were obtained 2–3 hours post surgery in the Ophthalmology Department (AOUP, University of Palermo, Italy). The rings were kept in Hank’s Balanced Salt Solution (HBSS; PAA, Pashing, Austria) and then cut into small segments to facilitate isolation of the limbus from the sclera.

### Enzymatic digestion and culture

The limbal segments were incubated with collagenase I (5 mg/ml; Sigma-Aldrich, St. Louis, MO, USA) overnight at 37 °C in a shaking bath. The following day, the digest was placed in a p60 dish culture (Corning, New York, USA) with the fibroblastic-maintenance medium (DMEM/F12 supplemented with 10 % embryonic stem cell-tested fetal bovine serum (EC-FBS; PAA), 1× ITS (5 μg/ml insulin, 5 μg/ml transferrin, 5 μg/ml selenium; PAA), and 20 ng/ml basic fibroblast growth factor (b-FGF; Preprotech, London, UK)) until cells reached confluence, changing the medium when necessary.

Subsequently, the f-LSC subculture was kept in the expansion medium (f-EM: DMEM/F12 supplemented with 5 % EC-FBS (PAA), 1× ITS (PAA), and 4 ng/ml b-FGF (Preprotech)).

### Doubling time

The f-LSCs were subcultured and seeded at a density of 4 × 10^3^ cells/cm^2^ and cell counts were performed at 24, 48, 72, 96, and 120 hours by optical microscope observation after staining with vital trypan blue. The doubling time was calculated online (http://www.doubling-time.com/compute.php; Roth V. 2006). For each sample of donor limbus, three sets of experiments were used for calculations.

### Sphere-forming assay

The f-LSCs were placed in ultralow-attachment six-well plates (Corning) at a density of no more than 1 × 10^2^ cells/cm^2^, and were cultured in f-EM without serum. Sphere formation was assessed by counting the number of spheres (cells > 3) under an optical microscope.

### Immunofluorescence staining

The spheres were transferred into a cell-culture chamber slide (Labtek II; Nunc, Waltham, MA, USA) and incubated in f-EM, under adhesion-condition culture at 37 °C in 5 % CO_2_ to allow their attachment. After 2 hours, cells were washed with phosphate-buffered saline (PBS) and fixed for 30 minutes in 2 % (wt/vol.) paraformaldehyde in PBS at room temperature, then incubated with 1 μg/ml PE-conjugated anti-human SSEA-4 monoclonal antibody (Miltenyi Biotec, Bergisch Gladbach, Germany) in PBS/bovine serum albumin (BSA; PAA) for 30 minutes, at room temperature. After incubation the cells were washed three times with PBS, counterstained with DAPI (Sigma Aldrich), and observed under a Zeiss Axiophot fluorescence microscope equipped with a Nikon DS-FI1 CCD camera.

Human bone marrow-derived MSCs (BM-MSCs; Lonza, Walkersville, MD, USA) were used as a stem marker positive control, HeLa (kindly obtained from Dr Salvatore Feo at University of Palermo) were used as an LESC marker positive control, and unstained f-LSCs were used as a negative control.

### Sorting assay through magnetic isolation

The f-LSCs were magnetically separated for SSEA4 expression by MACS MicroBead Technology (Miltenyi Biotec) according to the manufacturer’s instructions.

### Flow cytometry analysis

The cells were harvested and filtered through a 40-μm filter mesh and suspended at a concentration of 1 × 10^6^ cells/ml. Then 100 μl of cell suspension containing 5 × 10^5^ cells was used for each flow cytometric test.

#### Immunophenotyping

Human anti-HLA-DR, human anti-CD34, and human anti-CD45 monoclonal antibodies were tested on f-LSCs and were detected with the appropriate secondary antibody (Table 1). The incubation conditions were in accordance with the manufacturers’ instructions.

**Table 1:** Monoclonal antibodies used for the characterization of stem cell markers and cell phenotypes

Primary antibody/localization marker	Code number	Dilution	Incubation
HLA-DR, surface	Santa Cruz, sc-18875	1:50	o/n, r.t.
CD34, surface	Santa Cruz, sc-19621	1:50	o/n, r.t.
CD45, surface	Santa Cruz, sc-28369	1:50	o/n, r.t.
SSEA4, surface	Miltenyi Biotec, 130-98-371	1:100	30 minutes, r.t.
ABCG2, surface	BioLegend, 332002	1:100	o/n, r.t.
Δnp63, nuclear	BioLegend, 619002	1:100	o/n, r.t.
NANOG, surface	Santa Cruz, sc-293121	1:50	o/n, r.t.
OCT4, nuclear	Santa Cruz, sc-5279	1:50	o/n, r.t.
Secondary antibody	Code number	Dilution	Incubation
AlexaFluor 488	Life Technologies, Z25402	1:50	20 minutes, r.t.
AlexaFluor 594	Life Technologies, Z25007	1:50	20 minutes, r.t.

Miltenyi Biotec, Bergisch Gladbach, Germany

Santa Cruz, Dallas, Texas

BioLegend, London, UK

Life Technologies, Carlsbad, CA, USA

*o/n* overnight, *r.t.* room temperature

#### Stem cell phenotypes

The cells were double-stained with human anti-SSEA4 and human anti-ABCG2 monoclonal antibody, both surface MSC markers. The f-LSCs were tested for SSEA4 and for the human nuclear markers ΔNp63 or OCT4 or NANOG monoclonal antibody, after permeabilization with PBS supplemented with 0.1 % saponin and 1 % BSA for 20 minutes.

The antibody dilution, incubation, and detection conditions are presented in Table 1.

All reaction mixtures were then acquired with a FACS Calibur flow cytometer (Becton-Dickinson, Franklin Lakes, NJ, USA) and analyzed with the CellQuest Pro software. BM-MSCs were used as a positive control for SSEA4, NANOG, ABCG2, and OCT4, and HeLa cells were used as a positive control for ΔNp63.

### Analysis of cell cycle status of MSCs

Single cell suspensions of f-LSCs were obtained from two different culture passages: P4 and P30. For DNA content analysis, Nicoletti’s protocol was performed. Briefly, 1 × 10^6^ cells were fixed in 70 % ethanol, rehydrated in PBS, and then resuspended in a DNA extraction buffer (with 0.2 M NaHPO_4_, 0.1 % Tritonx-100, pH 7.8). After staining with 1 μg/ml of propidium iodide (PI) for 5 minutes, the fluorescence intensity was determined by analysis on a FACS Calibur flow cytometer (Becton-Dickinson). Data acquisition was performed with CellQuest software (Becton Dickinson), and the percentages of phase G1, S, and G2 cells were calculated with the MODFIT-LT software program (Verity Software House, Inc. Augusta, Topsham, ME, USA).

### RNA extraction, quantification, and retrotranscription

Total RNA was extracted and purified using E.Z.N.A. Total RNA Kit I (Omega Bio-Tek Inc., Norcross, GA, USA) according to the manufacturer’s instructions. RNA quantity and quality were assessed by Nano Drop 2000 (Thermo Scientific, Waltham, MA, USA), and 2 μg of f-LSC total RNA was reverse-transcribed to cDNA in a volume of 20 μl with Oligo dT primers (Applied Biosystems, Carlsbad, CA, USA) and the Reverse Transcriptase Rnase kit (Improm II; Promega, Madison, WI, USA).

### Real-time quantitative PCR

Real-time quantitative PCR primers were purchased from Qiagen (QuantiTect® Primer Assays; Qiagen, Milan, Italy) and Eurofin MWG (Biotech, Bergish Gladbach, Germany) and are listed in Table 2. All reactions were performed using the Quantitect SYBR Green PCR Kit (Qiagen, Valencia, CA, USA) on the RotorGene Q Instrument (Qiagen, Valencia, CA, USA). Each cDNA sample was mixed with specific primer sets and PCR master mix. The PCR parameters included denaturation at 95 °C for 3 minutes, then 40 cycles at 95 °C for 20 seconds, annealing at 60 °C for 30 seconds, and elongation at 72 °C for 60 seconds. Reactions were performed at least in triplicate. The specificity of the amplified products was determined by the melting peak analysis. The relative quantification model with efficiency correction was applied to normalize the expression of the target gene to β-actin (used as the housekeeping gene) and to compare gene expression with BM-MSCs (used as a positive cell control), on Rest2009 software (Relative Expression Software Tool; Qiagen, Valencia, CA, USA) [29]. The results were represented as histograms on GraphPad software (GraphPad Software, Inc., La Jolla, CA, USA).

**Table 2:** Real-time quantitative PCR primers used for gene expression investigation

Gene	Primer sequence	Code number
*CDKN1B*		QT00998445
*ABCG2*		QT00073206
*NANOG*		QT01844808
*OCT3*/4		QT00210840
*SOX2*	F-GGAGACGGAGCTGAAGCCGC	MWG
	R-GACGCGGTCCGGGCTGTTTT	
*THY*-1		QT00023569

### Protein extraction

The f-LSCs were scraped and incubated on ice for 30 minutes with M-RIPA buffer (50 mM Tris, pH 7.5, 0.1 % Nonidet P-40, 0.1 % deoxycholate, 150 mM NaCl, 4 mM EDTA) and a mixture of protease inhibitors (0.01 % aprotinin, 10 mM sodium pyrophosphate, 2 mM sodium orthovanadate, 1 mM PMSF). The whole cellular lysate was centrifuged at 12,000 rcf for 8 minutes to clear cell debris and the supernatant was dialyzed against ultrapure distilled water, lyophilized, and stored at –80 °C, as described previously [30, 31]. The protein concentration in the cellular extracts was determined using the Quick Start™ Bradford Protein Assay (BIO RAD, Segrate, Milan, Italy) according to the manufacturer’s instructions.

### Proteomic analysis

Two-dimensional gel electrophoresis (2D-IPG) was performed as described previously [30, 31]. Briefly, protein samples of f-LSCs were solubilized, and aliquots of 45 μg (analytical gels) or 1.5 mg (preparative gels) of total proteins were separately applied for isoelectrofocusing (IEF) using commercial sigmoidal IPG strips, 18 cm long with pH range 3.0–10; BIO RAD). The focused proteins were then separated on 9–16 % linear gradient polyacrylamide gels (SDS-PAGE) and were visualized by means of ammoniacal silver staining.

For image acquisition and data analysis*,* silver-stained gels were digitized using a computing densitometer and analyzed with ImageMaster 2D PLATINUM software (Amersham, Little Chalfont, Buckinghamshire, UK). Gel calibration was carried out using an internal standard and the support of the ExPaSy molecular biology server; the quantitative analysis of protein spots was performed in duplicate maps, and normalized as vol. % (integration of optical density over the spot area). The differential expression of proteins was evaluated when the difference in their values was ≥ 3 % volume. The labels correspond to the access number of the Swiss-Prot/TrEMBL database.

### Protein identification

Mass spectrometric sequencing was performed with the Voyager DE-PRO (Applied Biosystems, Carlsbad, CA USA) mass spectrometer [31]. Peptide mass fingerprinting was compared with the theoretical masses from the Swiss-Prot or NCBI sequence databases using Mascot (http://www.matrixscience.com/).

### Gene ontology and network analysis

The genes expressing the invariant and variant proteins were analyzed for their enrichment in specific gene ontology annotation using the Gene Ontology Consortium website (http://geneontology.org/) (Additional file 1: Table S2) [32]*.*

Network analysis was performed on the genes expressing the invariant and variant proteins using the STRING (Search Tool for the Retrieval of Interacting Genes/Proteins) website (http://string-db.org/) [33].

### Multilineage potential differentiation assays

Multilineage differentiation capability of f-LSCs at early and late passages (P4 and P20) was assessed. The following differentiation protocols were performed.

#### Osteogenesis

For osteogenic differentiation, 5 × 10^3^/cm^2^ f-LSC cells were cultured in home-made differentiation medium consisting of DMEM supplemented with 15 % FBS, 10^–4^ mM dexamethasone (Sigma-Aldrich), 10 mM glycerophosphate (Sigma-Aldrich), and 0.05 mM ascorbic acid (Sigma-Aldrich) [9]. After 21 days of culture in the differentiation medium, cells were stained with Alizarin red S (Sigma-Aldrich) to detect the calcium deposits. Briefly, the medium was removed and the cells were fixed with 4 % formaldehyde solution for 30 minutes, and after fixation were rinsed twice with distilled water and stained with 2 % Alizarin red S (pH 4.2) for 3 minutes. After incubation the cells were observed under a light optical microscope at 20–40× magnification.

#### Adipogenesis

Adipose differentiation was induced by seeding 5 × 10^3^/cm^2^ of f-LSCs and culturing in home-made differentiation medium consisting of DMEM medium containing 10 % FBS, 0.5 nM 1-methyl-3-isobutylxanthine (IBMX; Sigma-Aldrich), 10^–4^ mM dexamethasone, 10 μg/ml insulin, and 100 μM indomethacin (Sigma-Aldrich) [34]. To detect the presence of lipid droplets, cells were fixed with 4 % formaldehyde solution, rinsed twice in distilled water, stained with Oil Red O (Sigma-Aldrich), and observed under a light microscope at 20–40× magnification.

#### Chondrogenesis

Chondrogenic differentiation was induced by culturing cell mass in serum-free DMEM (PAA) with 10 ng/ml TGFβ-3 (Preprotech), 0.05 mM ascorbic acid (Sigma-Aldrich), 2 mM sodium pyruvate (PAA), and 10^–7^ mM dexamethasone (Sigma-Aldrich) in a six-well culture plate. Briefly, micromass cultures were generated by seeding 5-μl droplets of a cell solution of 1.6 × 10^7^ viable cells/ml in f-EM. After 2 hours of incubation at 37 °C, culture medium was replaced with chondrogenesis differentiation medium. After 21 days of culture the cell mass was evaluated morphologically and stained with Alcian blue to search for sulfated proteoglycan deposits.

### Statistical analysis

All assays were performed in triplicate. The data were reported as means ± SD and compared using the appropriate version of Student’s unpaired *t* test. Test results were reported as two-tailed *p* values, where *p* < 0.05 was considered statistically significant.

## Results

### Isolation of f-LSCs

**Fig. 1:**
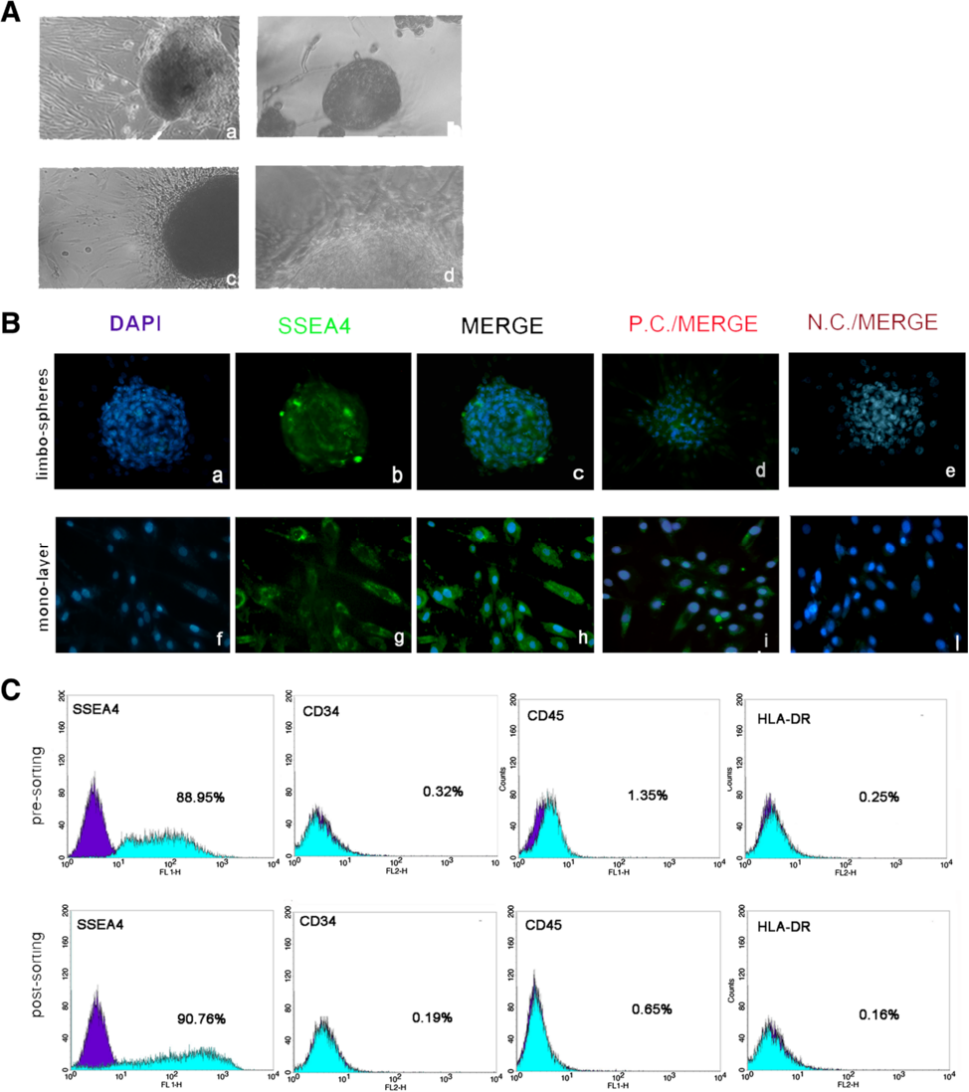
*A*: *a* Limbus digested after 2-week plate seeding: f-LSC growth in monolayer (P0 culture passage, 10×); *b* example of an f-LSC limbosphere in low-adhesion culture conditions (20×); *c, d* example of an f-LSC limbosphere under adhesion culture condition (*c* 20×; *d* 40×). *B*: SSEA4^+^ immunofluorescence staining: *a* DAPI on a limbosphere; *b* SSEA4 detection on a limbosphere (20×); *f* DAPI on monolayer; *g* SSEA4 detection in f-LSC monolayer (40×); *c, h* merge; *d, i* DAPI/SSEA4-stained BM-MSCs; *e*, *l* DAPI/SSEA4-stained HeLa cells. *C*: Cytofluorimetric assay in total population (presorting, *upper panel*) and in SSEA4^+^ f-LSCs (postsorting, *lower panel*). Cells are negative for CD34, CD45, and HLA-DR. All fields are representative of one limbus sample out of at least 12 independent experiments. *DAPI* 4′,6-diamidino-2-phenylindole, *N.C.* negative control, *P.C.* positive control

We determined that approximately 3 × 10^4^–5 × 10^4^ cultured f-LSCs were isolated from each explanted human corneo-scleral ring after enzymatic digestion. The digests were kept in culture in f-EM (we referred to these culture passages as P0) and in 2 weeks a single cell population with an elongated shape was obtained (Fig. 1A.a).

Within 10 days the cells, kept in a low-adhesion culture condition, gave rise to floating spherical cell bodies (considered a hallmark of the stemness feature) with a clear and well-delineated border, which we referred to as “limbospheres” (Fig. 1A.b). When transferred to adhesion conditions the limbospheres spread out and a monolayer culture of fibroblast-like cells formed (Fig. 1A.c,d).

SSEA4 expression was investigated by immunofluorescence analysis both in f-LSC spheres (Fig. 1B.a–c) and in monolayer f-LSCs (Fig. 1B.f–h). BM-MSCs and HeLa cells were respectively used as positive control (Fig. 1B.d–i) and negative control (Fig. 1B.e–l). As reported in the literature, it did not seem possible to obtain spheres from cells that have no SSEA4 expression.

Flow cytometry demonstrated that SSEA4 was highly expressed (88.95 ± 7.8 %) in f-LSC monolayer cultures and did not significantly increase after sorting (90.76 ± 5.6 %). Immunophenotypes were analyzed in the presorting (total) population and the postsorting SSEA4^+^ population. Both populations showed almost no expression of CD34 (0.32 ± 0.01 % vs. 0.19 ± 0.02 %, respectively), CD45 (1.35 ± 0.7 % vs. 0.65 ± 0.2 %, respectively), and HLA-DR (0.25 ± 0.04 % vs. 0.16 ± 0.07 %, respectively) (Fig. 1C).

Since neither population displayed any significant differences in marker expression, the subsequent cell analyses were only performed on the total monolayer f-LSC population.

### Proliferation in long-term culture

**Fig. 2:**
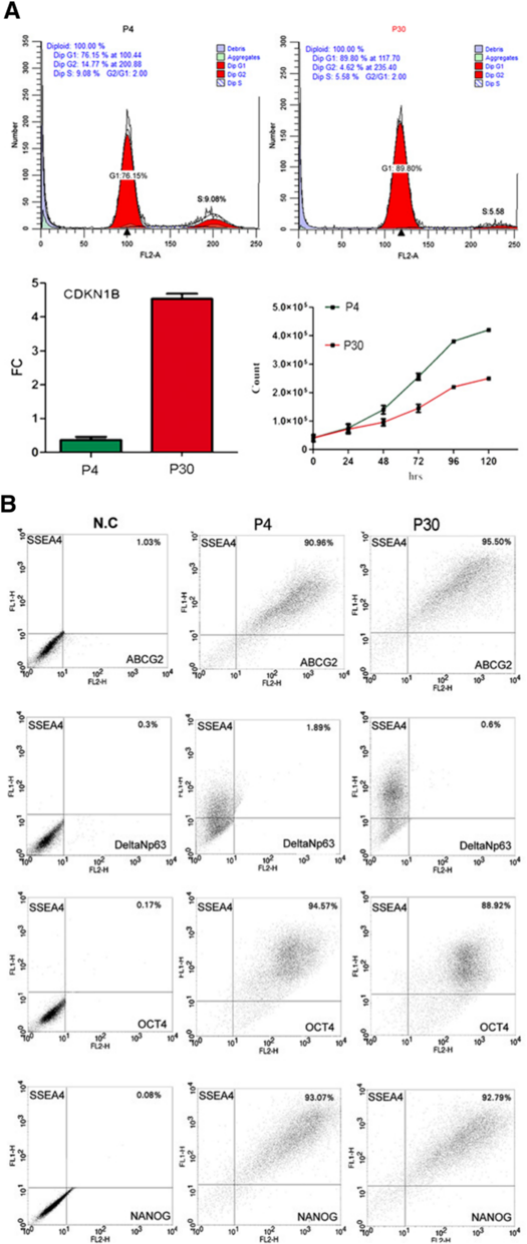
**a** (*Upper panel*) Cell cycle distribution of f-LSCs (P4 on the *left*; P30 on the *right*) performed according to Nicoletti’s protocol. (*Lower panel*) Increment of expression of CDKN1B in P30 vs*.* P4 (*left*); kinetics of f-LSCs at early passage (P4) and late passage (P30) in expansion medium (*right*). Mean values ± SD of a set of 25 experiments after 6 days. **b** Cytometric detection of double-positive cell populations for pluripotent stem cell markers at two different culture passages: early passage (P4) and late passage (P30). *P* passage

The distribution of f-LSCs in the various stages of the cell cycle was investigated at two different passages. The early passage P4 showed an increase in the percentage of cells arrested in the G1 phase compared with the late-passage P30 (76.15 ± 2.45 % vs. 89.90 ± 3.65 %, *p* < 0.05) and a decrease in the percentage of cells in the S phase and in the G2 phase (Fig. 2a, upper panel). Moreover, in P30 the mRNA level of CDKN1B (cyclin-dependent kinase inhibitor 1B, also known as P37) was increased up to 4.59-fold relative to P3 (Fig. 2a, left-lower panel). The culture doubling time was slightly extended in P30 with respect to P3 f-LSCs, reaching about 46.2 ± 5.37 hours vs. 39.6 ± 2.54 hours (Fig. 2a, right-lower panel).

### Flow cytometry stem cell phenotype characterization in long-term culture

Phenotype characterization was performed at two different culture passages (P3 and P30). A collection of double-positive populations (dpp) was obtained for several stem markers. The data expressions detected with flow cytometry analysis are the following (P3 vs. P30): ABCG2^+^/SSEA4^+^ = 98.6 ± 7.1 % vs. 96.6 ± 4.7 % (*p* = NS); OCT4^+^/SSEA4^+^ = 92.1 ± 6.4 % vs. 88.3 ± 2.6 % (*p* = NS); and NANOG^+^/SSEA4^+^ = 95.42 ± 6.8 % vs. 80.52 ± 4.2 % (*p* = NS). The f-LSCs did not express Δnp63 (Δnp63^+^/SSEA4^+^ < 0.5 %). Figure 2b shows one representative experiment out of a total of 25 sets of experiments.

The positive controls are shown in Additional file 2: Figure S1.

### Stem-related gene expression is not influenced by age and by long-term cultures

**Fig. 3:**
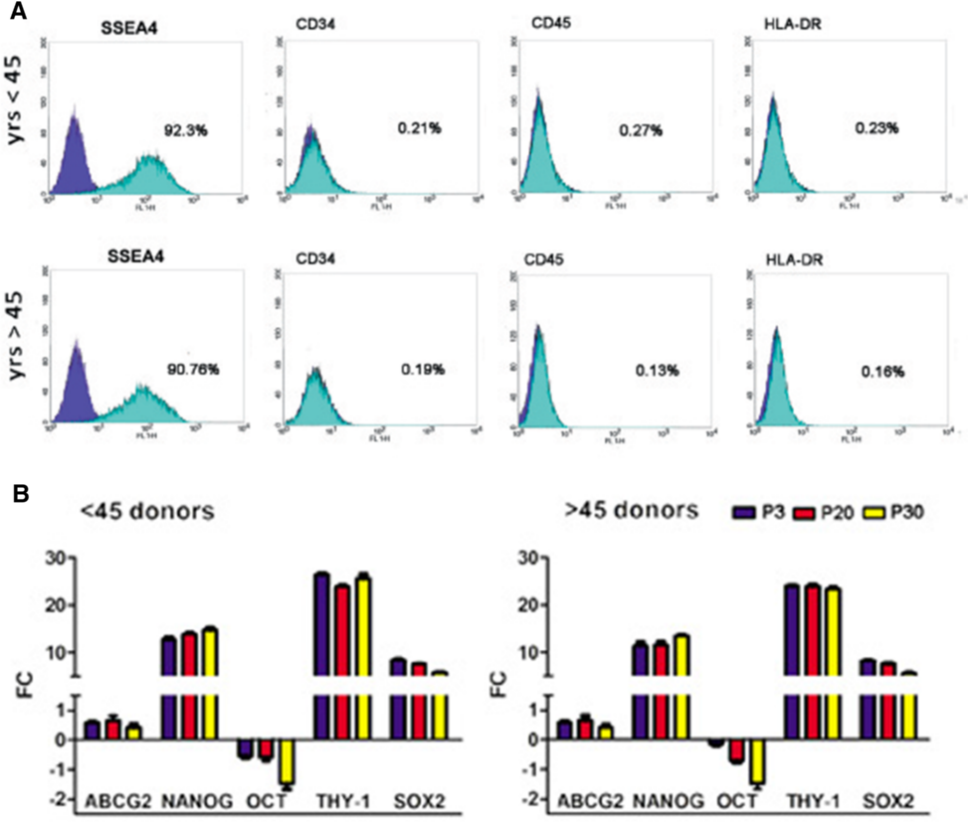
Donor age and long-term culture effect on stem cell profile. **a** f-LSCs maintain negativity for CD34, CD45, and HLA-DR and are highly positive for SSEA4 in patients < 45 years old (*upper graph*) and > 45 years old (*lower graph*). **b** Real-time quantitative PCR analysis of pluripotent stem cell markers in f-LSC different culture passage obtained from patients of different ages. Mean values ± SD of all limbus donors studied are reported. *P* passage

We compared the f-LSC stem molecular expression pattern in young donors (age < 45 years: *N* = 11, six males and five females; Group A) and older donors (age > 45 years: *N* = 14, eight males and six females; Group B). No substantial differences were found in the immune phenotype (Fig. 3a): both Groups A and B were negative for CD34 (0.21 ± 0.07 % vs. 0.19 ± 0.1 %, *p* = NS), CD45 (0.27 ± 0.1 % vs. 0.13 ± 0.08 %, *p* = NS), and HLA-DR (0.23 ± 0.12 % vs. 0.16 ± 0.04 %, *p* = NS) whereas they highly expressed SSEA4 (92.3 ± 5.1 % vs. 90.76 ± 4.61 %, *p* = NS). The expression analysis performed on three different culture passages (P3, P20, and P30) showed no substantial difference for all stem cell markers tested between f-LSCs of patients within the two different groups (Figs. 3b,c). Comparing the gene expression levels of the two groups, it was clear that there was no significant age-related difference. The f-LSC stem cell markers, NANOG, THY-1, and SOX-2, were firmly maintained, as well as ABCG2, thus confirming maintenance of the limbal nature. Only OCT4 relative expression showed a faint decrease that appeared to be correlated with long-term culture but not with age. Moreover, CK12 (cytokeratin 12, corneal epithelial marker) and ΔNp63 (LESC marker) were not expressed (or only weakly expressed; data not shown), thus confirming that the culture conditions were not suitable for epithelial differentiation. Our findings showed that cultured and expanded f-LSCs exhibited similar expression patterns both in long-term culture and soon after isolation, maintaining their own expression characteristics.

### The f-LSC proteomic profile is not influenced by age and by long-term cultures

**Fig. 4:**
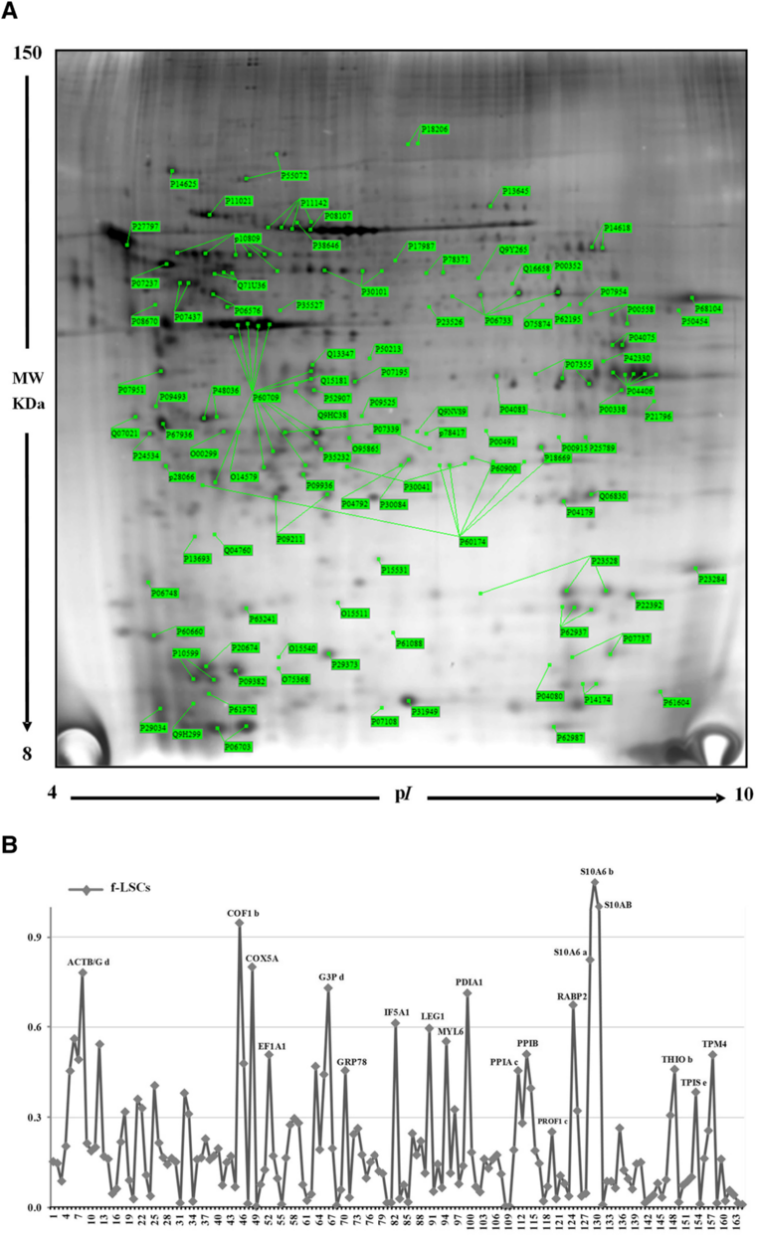
**a** f-LSC two-dimensional electrophoresis master proteomic map with labeled proteins. Two-dimensional gel electrophoresis assay resulted in a master gel that revealed 164 spots, 78 % of which had an average pixel density of about 0.3. **b** Densitometry profile of f-LSC proteins*. f-LSC* fibroblast-like limbal stem cell

To further characterize the f-LSC phenotype, we performed a proteomic analysis. Two-dimensional gel electrophoresis assay resulted in a master gel that revealed 164 spots, 78 % of which had an average pixel density of about 0.3 (Fig. 4a). The list of assigned proteins is shown in Additional file 3: Table S1. The derived protein f-LSC profiles represented by the densitometry, expressed as a graph, are shown in Fig. 4b, which also shows the relative intensities of the single protein expression levels. In Additional file 4: Figure S2, panels A and B show the proteomic maps of f-LSCs from the same older donor (Group B) at two different culture passages (P4 vs. P20), panels C and D show the proteomic maps of f-LSCs from two different young donors of the same age (Group A) at the same passage culture, and panel E shows the proteomic map of P20 f-LSCs from one 64-year-old donor (Group B). By comparing the f-LSC P4/B and f-LSC P20/B proteomic profiles, we detected overlapping for about 90 % of the proteins expressed (Additional file 5: Figure S3A); to test the occurrence of possible individual variants we compared the proteomic profile of f-LSCs from different donors of the same age (Group A) and we found overlapping for about 83 % of the proteins (Additional file 5: Figure S3B). Moreover, comparing the proteomic profiles of Group A with Group B donor at the same culture passage, the proteomic profiles overlapped by about 68 % (Additional file 5: Figure S3C).

**Fig. 5:**
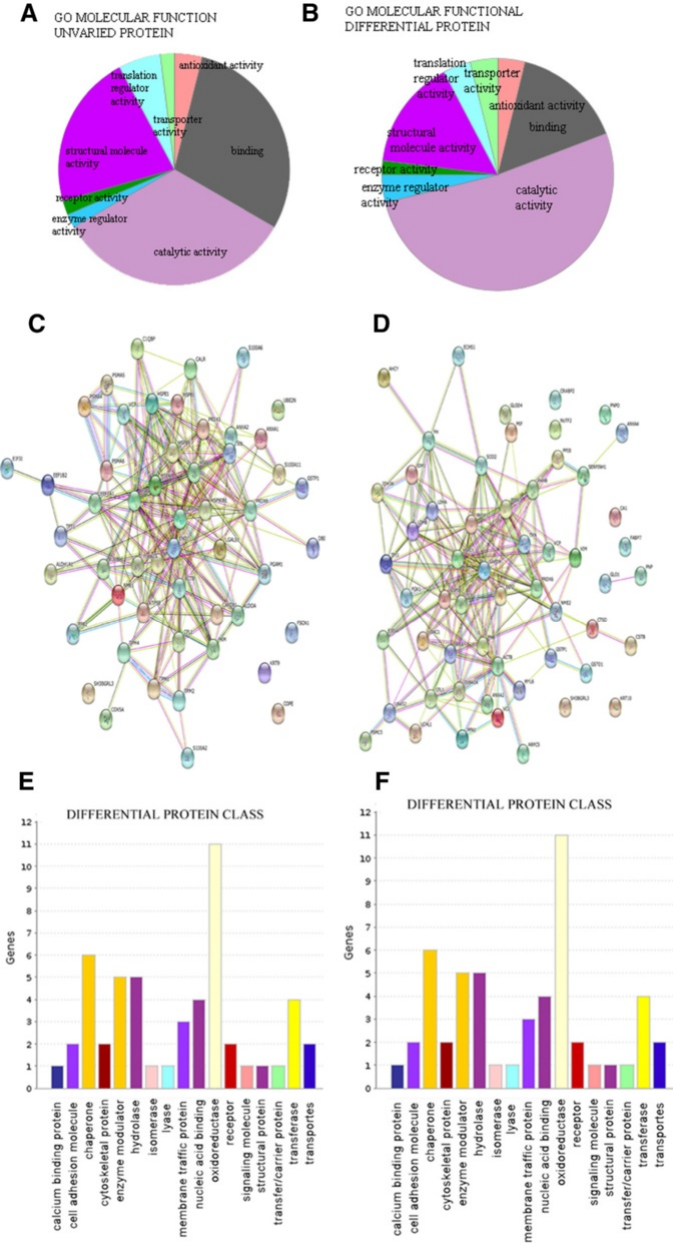
Pie charts representing the GO molecular function of unvaried proteins **a** and differential expressed proteins **b**. Protein network of f-LSC unvaried proteins **c** and differential expressed proteins, performed on the STRING website **d**. Protein class distribution of unvaried **e** and differential expressed proteins **f**, performed on the Gene Ontology website

By comparative analysis between the proteomic profiles of all investigated conditions, a protein group with higher expression levels was detected. Eighty of the proteins maintained invariant expression levels, whereas 84 proteins proved to be differentially expressed (Additional file 1: Table S2). The Gene Ontology analysis showed that they were related to cell growth/maintenance and apoptosis, energy pathways and signal transduction, membrane protein metabolism, protein biosynthesis, folding and degradation, cytoskeleton, and motility (Additional file 6: Table S3 and S4). Both in the invariant protein group and the differential protein group the “catalytic activity” was the most abundant class (33.3 % vs. 52.9 %, respectively). The “structural molecule activity” was also well represented (21.6 % vs.13.7 %*,* unvaried vs*.* differential proteins) (Fig. 5a,b) [34–46].

The protein-interaction networks (PIN) for the unvaried protein groups revealed strong interactions between structural proteins (Fascin-1, cytoskeletal keratin 19), chaperones, including two heat shock proteins (HSP70, HSP90), DNA binding protein (i.e., elongation factors EEF1A, EEF1B2) and remodeling and proteosome complex (PSMA5,4,6). This profile is consistent with a stem phenotype that requires plasticity and good proliferative ability [47, 48].

Interestingly, the hypoxia-inducible factor-1 (HIF-1) pathway is a central node of differential protein group PIN with close correlation between superoxide dismutase 2 (SOD2), thioredoxin (TXN), peroxiredoxin 6 (PRDX6), and catepsin D (CTSD). It has been shown that HIF-1 is involved in self-renewal and maintenance of pluripotency, by promoting the expression of the putative stemness genes (OCT4, SOX2, NANOG, Klf4) [49–51].

### Multilineage capability of f-LSCs

**Fig. 6:**
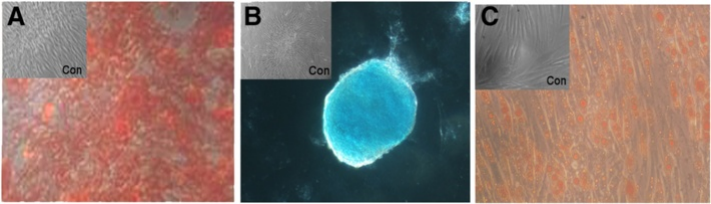
**a** f-LSCs stained with Alizarin red S for detection of calcific deposition in cultured f-LSCs for differentiation towards an osteogenic lineage. **b** Alcian blue staining to detect chondrogenic differentiation. **c** Oil Red O-stained neutral triglycerides and lipids on adipose-differentiated cells. The appropriate medium for the multilineage differentiation is reported in Methods

We tested the differentiation capability of f-LSCs at an early passage (P3) and a late passage (P10). Both were differentiated in vitro using osteoblastic, adipose, and chondrogenic home-made media, as described in Methods. Twenty-one days after osteoblastic induction, Alizarin red S revealed the presence of extracellular matrix mineralization in both f-LSC cultures suggesting osteoblastic differentiation (Fig. 6a). lcian blue positivity confirmed the presence of acid polysaccharides (such as glycosaminoglycans) suggesting chondrogenic differentiation 21 days after induction (Fig. 6b).

Moreover, oil red positivity confirmed the presence of neutral triglyceride and lipid vacuoles, suggesting adipogenic differentiation after 28 days of culture (Fig. 6c).

## Discussion

Ease of access and immune-privileged status make the eye an ideal organ for its potential application in regenerative medicine. In our study, we confirmed the ease of isolation of the subpopulation of f-LSCs, which make them ideal candidates for adult stem cell therapy. In spite of the great interest in clinical applications, no exhaustive information is yet available on f-LSCs, especially on their exact characteristics. The International Society for Cellular Therapy has defined criteria to identify MSCs [52]. We adopted these criteria to characterize f-LSCs from the limbus in our study. In addition, this work provides convincing evidence that limbal stroma contains f-LSCs. The latter subpopulation resembles stem cells which reside in the stem cell niche, where they are maintained in an undifferentiated state. These observations were supported by Notara and Daniels [53] who showed that LESCs are discretely located in the basal layer of the corneal limbal epithelium, at the junction between the transparent cornea and the opaque sclera. The limbal palisades of Vogt have been proposed as the site of the LESC niche whereas f-LSCs seem to reside in the extracellular matrix [3, 4]. Corneal stromal stem cells have been located in the anterior stroma subadjacent to the basal side of the palisades of Vogt [54–60]. In our study we did not aim to define the exact localization of f-LSCs but confirmed that they are completely different from LESCs, as demonstrated by ΔNp63 and CK12 and CK19 negativity, and they can be easily isolated and grown.

In our research we showed that f-LSCs fulfilled the criteria for multipotency established today.

In particular, our group investigated the possible effects of long-term culture conditions and donor age on the f-LSC phenotype. The cells isolated did not display surface expression of any hematopoietic marker (CD34 and CD45) and HLA-DR. By contrast, they expressed a variety of stemness markers (SSEA4, ABCG2, OCT3/4, and NANOG).

They showed low adhesion growth capability as limbo-spheres and they should proliferate in vitro as adherent cells for long-term culture, maintaining positivity for ABCG2, OCT3/4, NANOG, SOX2, THY-1, and SSEA4 and negativity for CD34, CD45, and HLD-DR; and they have multilineage differentiation potential under in-vitro culture conditions. By comparing the cell cycle distribution and the proliferation curve in the early and late passages there was a weak increase in the percentage of cells in phase G1 with a concomitant decrease in the percentage of cells in phase S. This event is reflected as a slight elongation in the doubling time in late passage culture but was not analyzed in more detail.

The evaluation of molecular expression of f-LSCs isolated from different donors (with the same or different age) and at different culture passages revealed a strong stability of the “limbal stem molecular pattern”, not affected by long-term culture and donor age. No significant differences were found regarding specific osteogenic, adipogenic, or chondrogenic staining.

For the first time, we constructed a two-dimensional electrophoresis (2-DE) proteomic pattern of cultured f-LSCs. The derived pattern of resolved protein spots was highly consistent; we identified 164 proteins stably expressed in the different conditions, defining profiles enriched in proteins linked to cell plasticity and proliferative and self-renewal capability. Furthermore, overlapping protein expression profiles confirm the stability of the stem cell phenotype with higher expression of structural proteins and proteins involved in the stem molecular pathway.

## Conclusions

f-LSCs represent a robust source of MSCs independently of donor age and in-vitro conditions; the stability of their proteomic pattern could be very promising, suggesting their potential use in regenerative medicine.

## Abbreviations

ABCG2, ATP-binding cassette sub-family G member 2; BM-MSC, bone marrow-derived mesenchymal stromal cell; f-LSC, fibroblast-like limbal stem cell; LESC, limbal epithelial stem cell; MSC, mesenchymal stromal cell; NANOG, Homeobox protein NANOG; OCT4, octamer-binding transcription factor 4; P, passage of culture; PBS, phosphate-buffered saline; PIN, protein-interaction networks; SOX2, SRY (sex determining region Y)-box 2; SSEA4, stage-specific embryonic antigen-4; ΔNp63, a splice variant of p63

## Additional files

Additional file 1: Table S2 Catalogue of the unvaried and differential expressed proteins in f-LSCs samples. (ZIP 110 kb) Additional file 2: Figure S1 Positive Control of cytofluorimetric assay: Human bone marrow mesechymal stem cells (BM-MSCs) stained positive for SSEA4 (A), ABCG2 (B), OCT4 ( C ) and NANOG (D); HeLa cells stained positive for ΔNp63 (E). A cell suspension of BM-MSCs and HeLa was obtained by trypsinization and centrigugation. The cell pellet was resuspended in 100μl of PBS to test SSEA4 or ABCG2. The samples were resuspended in PBS supplemented with 0.1% saponin and 1% BSA for 20 minutes for permeabilization cells and finally stained for OCT4 or NANOG or ΔNp63. (TIF 686 kb) Additional file 3: Table S1 Catalogue of the protein spots identified in the 2D-IPG prototype map of f-LSCs. Table reports the following information: Protein names, accession numbers (AC) and abbreviated names correspond to the nomenclature used in the Swiss-Prot database. The experimental values of pI and MW for every isoelectric spot were calculated with ImageMaster 2D Platinum system; the theoretical values represent the predicted MW and pI for each identified protein according to Swiss-Prot and TrEMBL database. Identification methods: 1, MALDI-TOF; 2, N-terminal sequencing by automated Edman degradation and 3, Western Blotting. (DOC 198 kb) Additional file 4: A) The proteomic map of P4 f-LSCs from >45 years donor; B) The proteomic map of P20 f-LSCs from >45 years donor f-LSCs; C, D) The proteomic maps of P10 f-LSCs from 2 donors < 45 years; E) The proteomic map of P20 f-LSCs from one 64 years old donor. (TIF 2807 kb) Additional file 5: Figure S3 Figure.5: A) Proteomic profile analysis of P4 f-LSCs>45 years vs. f-LSC P20 f-LSCs >45 years, overlapped for about 90% of proteins expressed; B) f-LSCs from 2 different donors of the same age (<45 years) overlapped for about 83% of proteins; C) proteomic profiles of the same cutlure passage f-LSCs from 2 donors of different ages (< 45 years vs. >45 years) overlapped for about 68%. P= passage. (TIF 25509 kb) Additional file 6: Table S3. The genes expressing the invariant proteins were analyzed for their enrichment in specific gene ontology annotation. Table S4. The genes expressing the differenzial proteins were analyzed for their enrichment in specific gene ontology annotation. (XLS 16 kb)
