# Erratum to: Donor age and long-term culture do not negatively influence the stem potential of limbal fibroblast-like stem cells

**DOI:** 10.1186/s13287-016-0381-5

**Published:** 2016-08-10

**Authors:** Laura Tomasello, Rosa Musso, Giovanni Cillino, Maria Pitrone, Giuseppe Pizzolanti, Antonina Coppola, Walter Arancio, Gianluca Di Cara, Ida Pucci-Minafra, Salvatore Cillino, Carla Giordano

**Affiliations:** 1 Laboratory of Regenerative Medicine, Section of Endocrinology, Diabetology and Metabolism, Di.Bi.M.I.S., University of Palermo, Piazza delle Cliniche 2, 90127 Palermo, Italy; 2 Centro di Oncobiologia Sperimentale (COBS), Palermo, Italy; 3 Department of Ophthalmology, University of Palermo, Palermo, Italy; 4 ATeN (Advanced Technologies Network Center), University of Palermo, Palermo, Italy

## Erratum

Following publication of the original article in *Stem Cell Research & Therapy* [1], it was brought to our attention that panel 5E in Fig. 5 is a duplicate of panel 5F. Please find below the figure with the correct panel E. We apologize for the inconvenience this may have caused.

**Fig. 5:**
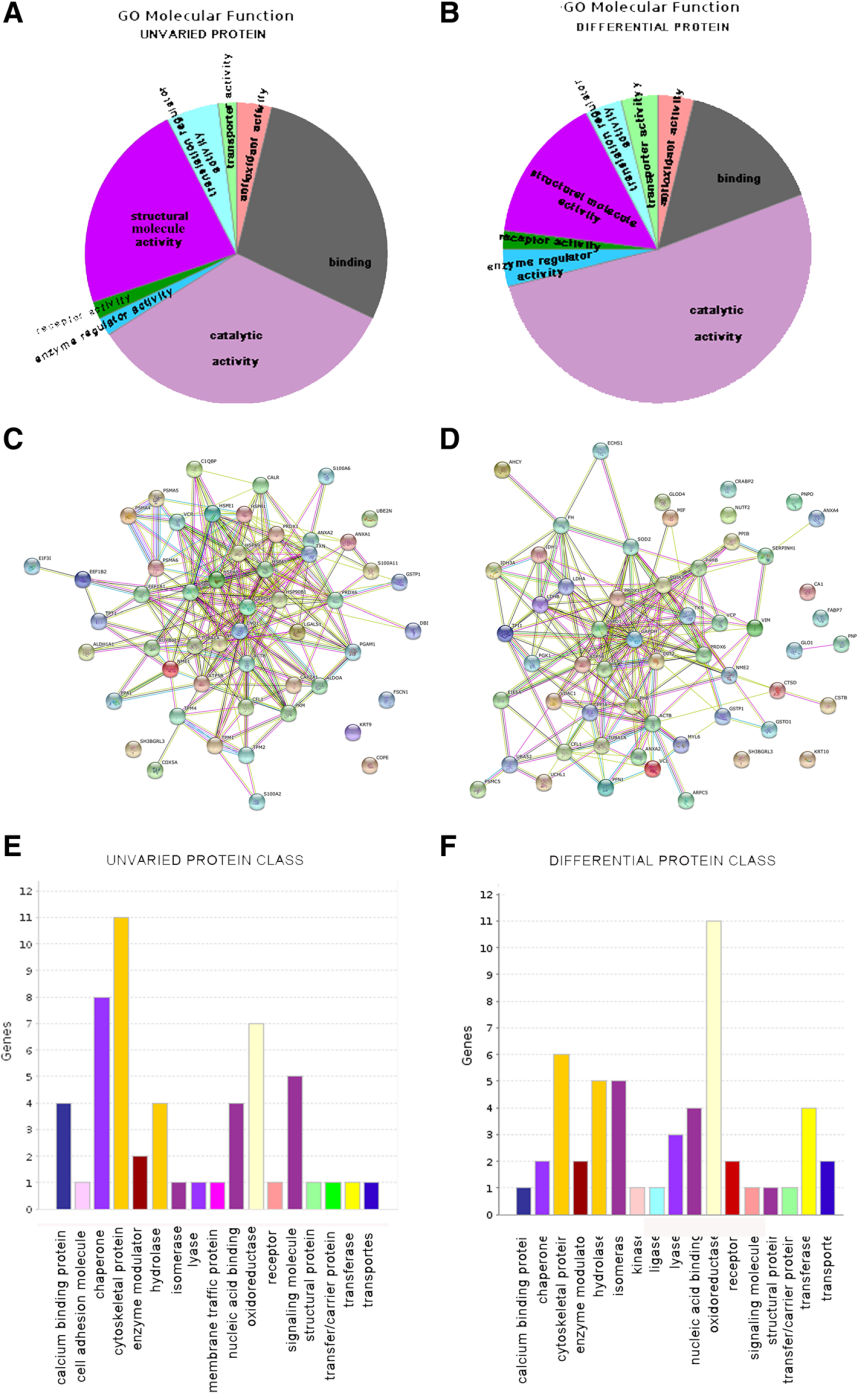
Pie charts representing the GO molecular function of unvaried proteins **a** and differential expressed proteins **b**. Protein network of f-LSC unvaried proteins **c** and differential expressed proteins, performed on the STRING website **d**. Protein class distribution of unvaried **e** and differential expressed proteins **f**, performed on the Gene Ontology website
