# Supplementary figures and images for: Donor age and long-term culture do not negatively influence the stem potential of limbal fibroblast-like stem cells
Source: Stem Cell Res Ther. 2016 Jun 13;7:83. doi: 10.1186/s13287-016-0342-z (PMC4906894; doi:10.1186/s13287-016-0342-z)

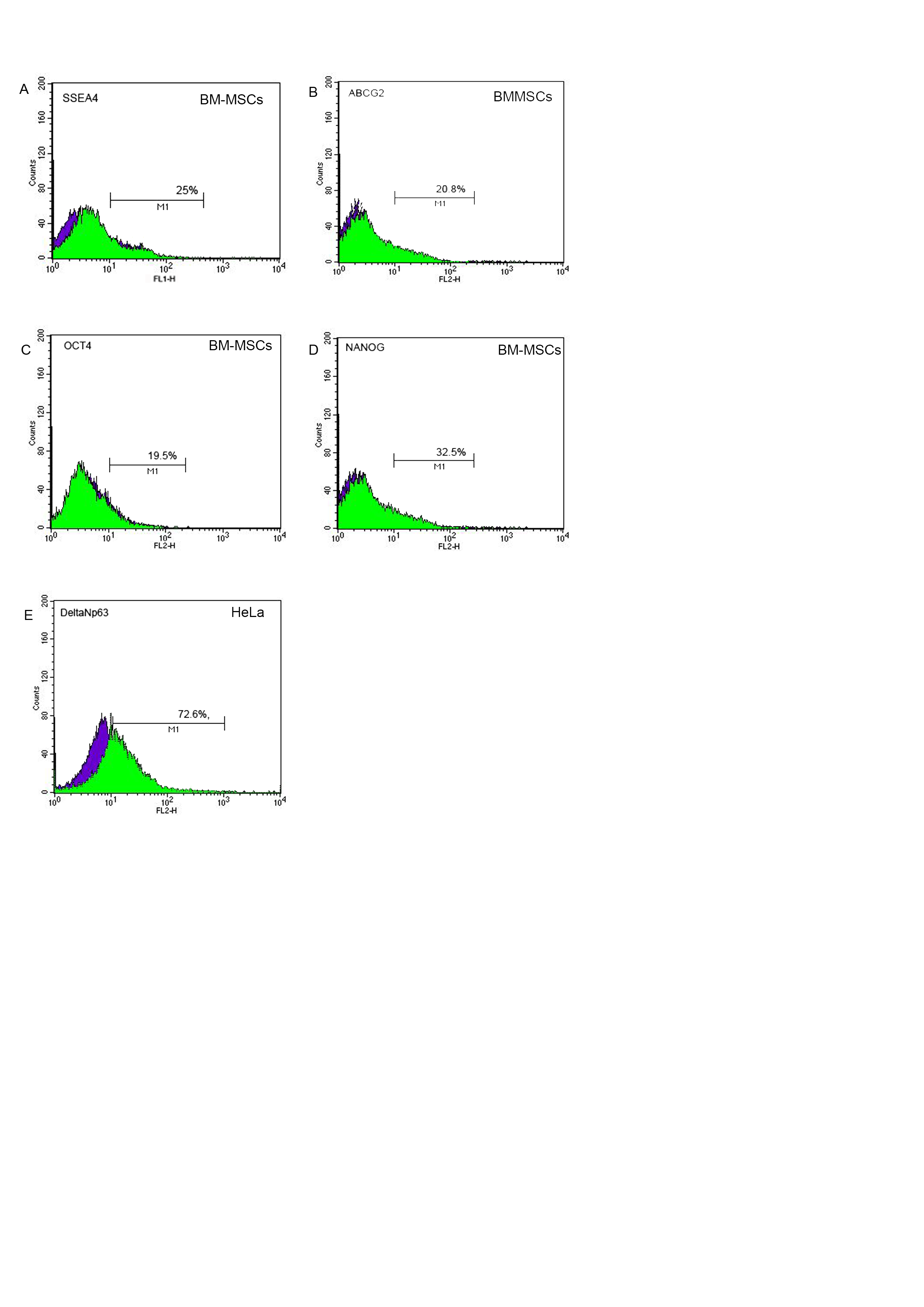

Supplement: Additional file 2: — Figure S1 Positive Control of cytofluorimetric assay: Human bone marrow mesechymal stem cells (BM-MSCs) stained positive for SSEA4 (A), ABCG2 (B), OCT4 ( C ) and NANOG (D); HeLa cells stained positive for ΔNp63 (E). A cell suspension of BM-MSCs and HeLa was obtained by trypsinization and centrigugation. The cell pellet was resuspended in 100μl of PBS to test SSEA4 or ABCG2. The samples were resuspended in PBS supplemented with 0.1% saponin and 1% BSA for 20 minutes for permeabilization cells and finally stained for OCT4 or NANOG or ΔNp63. (TIF 686 kb) [file 13287_2016_342_MOESM2_ESM.tif]

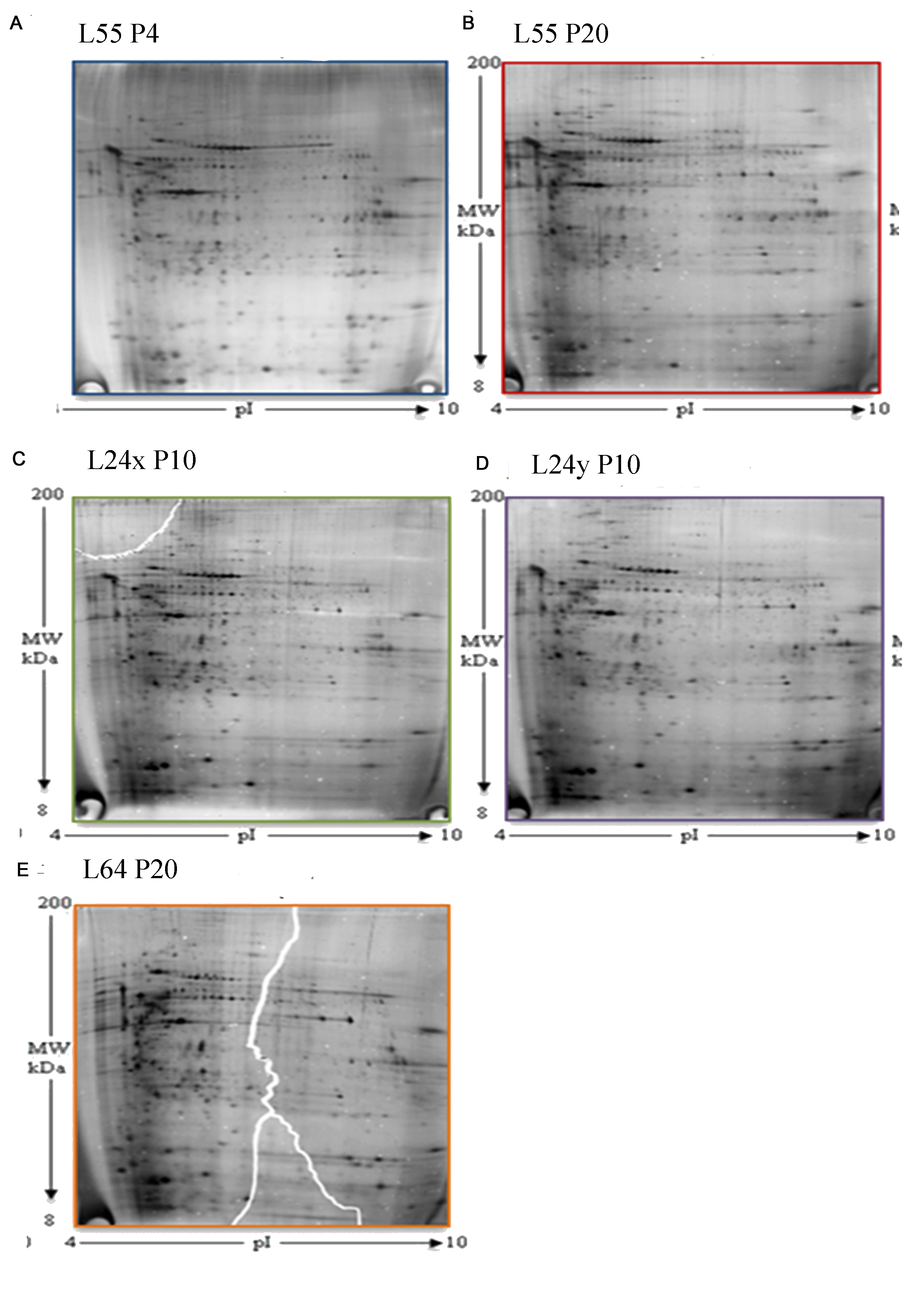

Supplement: Additional file 4: — A) The proteomic map of P4 f-LSCs from >45 years donor; B) The proteomic map of P20 f-LSCs from >45 years donor f-LSCs; C, D) The proteomic maps of P10 f-LSCs from 2 donors < 45 years; E) The proteomic map of P20 f-LSCs from one 64 years old donor. (TIF 2807 kb) [file 13287_2016_342_MOESM4_ESM.tif]

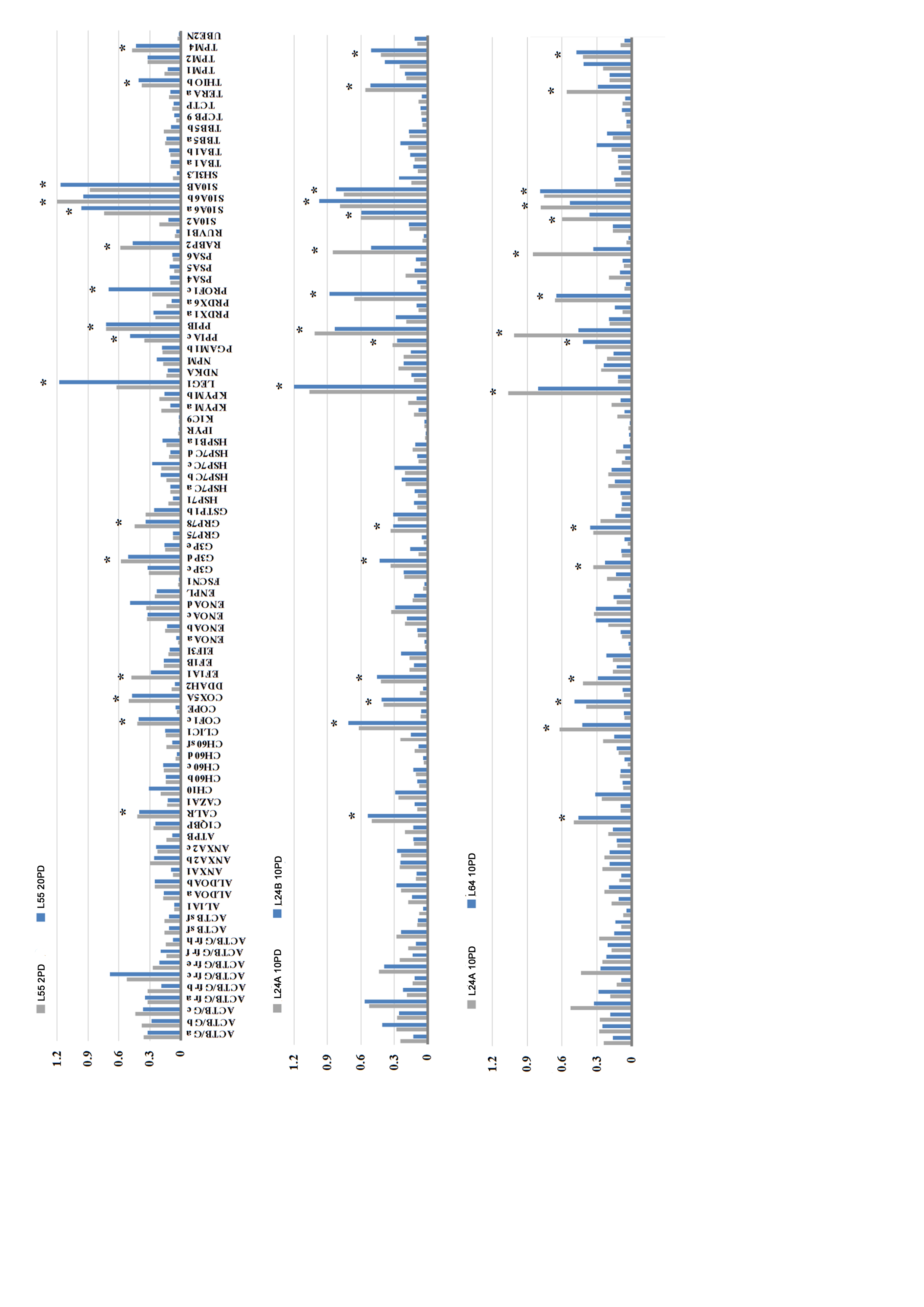

Supplement: Additional file 5: — Figure S3 Figure.5: A) Proteomic profile analysis of P4 f-LSCs>45 years vs. f-LSC P20 f-LSCs >45 years, overlapped for about 90% of proteins expressed; B) f-LSCs from 2 different donors of the same age (<45 years) overlapped for about 83% of proteins; C) proteomic profiles of the same cutlure passage f-LSCs from 2 donors of different ages (< 45 years vs. >45 years) overlapped for about 68%. P= passage. (TIF 25509 kb) [file 13287_2016_342_MOESM5_ESM.tif]
