# Supplementary material for: Donor age and long-term culture do not negatively influence the stem potential of limbal fibroblast-like stem cells
Source: Stem Cell Res Ther. 2016 Jun 13;7:83. doi: 10.1186/s13287-016-0342-z (PMC4906894; doi:10.1186/s13287-016-0342-z)
Supplement: Additional file 3: — Table S1 Catalogue of the protein spots identified in the 2D-IPG prototype map of f-LSCs. Table reports the following information: Protein names, accession numbers (AC) and abbreviated names correspond to the nomenclature used in the Swiss-Prot database. The experimental values of pI and MW for every isoelectric spot were calculated with ImageMaster 2D Platinum system; the theoretical values represent the predicted MW and pI for each identified protein according to Swiss-Prot and TrEMBL database. Identification methods: 1, MALDI-TOF; 2, N-terminal sequencing by automated Edman degradation and 3, Western Blotting. (DOC 198 kb) [file 13287_2016_342_MOESM3_ESM.doc]

Additional Table.1

| ***** | **Abbreviated Name** | **Protein Name** | **AC Number** | **Theor MW** | **Exp MW** | **Theor pI** | **Exp pI** | **ID methods** | **% Masses Matched** | **Sequence coverage (%) N-terminal residues** |
| --- | --- | --- | --- | --- | --- | --- | --- | --- | --- | --- |
| **1** | ACBP | Acyl-CoA-binding protein | P07108 | 10044 | 10061 | 6.12 | 5.84 | 1 | 17 | 50 |
| **2** | ACTB/G a | Actin, cytoplasmic 1 | P60709 | 41737 | 42144 | 5.29 | 5.08 | 1,3 | 36 | 22 |
| **3** | ACTB/G b |  |  |  | 42432 |  | 5.13 | 1,3 | 33 | 23 |
| **4** | ACTB/G c |  |  |  | 42000 |  | 5.18 | 1,3 | 14 | 31 |
| **5** | ACTB/G d |  |  |  | 42288 |  | 5.24 | 1,3 | 45 | 41 |
| **6** | ACTB/G fr a |  |  |  | 36378 |  | 5.46 | 2 | - | res.62-71 |
| **7** | ACTB/G fr b |  |  |  | 35440 |  | 5.46 | 1 | 21 | 34 |
| **8** | ACTB/G fr c |  |  |  | 29416 |  | 5.49 | 1 | 39 | 30 |
| **9** | ACTB/G fr d |  |  |  | 28354 |  | 5.49 | 1 | 26 | 27 |
| **10** | ACTB/G fr e |  |  |  | 27532 |  | 5.26 | 1 | 24 | 22 |
| **11** | ACTB/G fr f |  |  |  | 26669 |  | 5.43 | 1 | 52 | 46 |
| **12** | ACTB/G fr g |  |  |  | 26669 |  | 4.96 | 1 | 55 | 16 |
| **13** | ACTB/G fr h |  |  |  | 26669 |  | 5.21 | 1 | 22 | 26 |
| **14** | ACTB/G sf |  |  |  | 40518 |  | 5.04 | 1 | 22 | 29 |
| **15** | AK1C3 | Aldo-keto reductase family 1 member C3 | P42330 | 36853 | 37540 | 8.06 | 6.81 | 1 | - | 30 |
| **16** | AL1A1 | Retinal dehydrogenase 1 | P00352 | 54862 | 53159 | 6.30 | 6.83 | 1 | 61 | 49 |
| **17** | ALDOA a | Fructose-bisphosphate aldolase A | P04075 | 39420 | 39529 | 8.30 | 6.92 | 1 | 62 | 59 |
| **18** | ALDOA b |  |  |  | 39529 |  | 7.10 | 1 | 40 | 20 |
| **19** | ANXA1 | Annexin A1 | P04083 | 38714 | 35789 | 6.57 | 6.52 | 1 | 46 | 52 |
| **20** | ANXA2 a | Annexin A2 | P07355 | 38604 | 36053 | 7.57 | 6.74 | 1 | 21 | 20 |
| **21** | ANXA2 b |  |  |  | 35527 |  | 6.88 | 1 | 50 | 15 |
| **22** | ANXA2 c |  |  |  | 34837 |  | 6.84 | 1 | 50 | 15 |
| **23** | ANXA2 sf |  |  |  | 31199 |  | 6.87 | 1 | 54 | 30 |
| **24** | ANXA4 | Annexin A4 | P09525 | 35883 | 31122 | 5.83 | 5.73 | 1 | 36 | 64 |
| **25** | ANXA5 a | Annexin A5 | P48036 | 35752 | 30894 | 4.82 | 4.90 | 1 | 29 | 28 |
| **26** | ANXA5 b |  |  |  | 31046 |  | 4.96 | 1 | 59 | 65 |
| **27** | ARPC5 | Actin-related protein 2/3 complex subunit 5 | O15511 | 16320 | 24150 | 5.47 | 5.60 | 1 | 69 | 48 |
| **28** | ATPB | ATP synthase subunit beta, mitochondrial | P06576 | 56560 | 48976 | 5.26 | 4.95 | 1 | 89 | 59 |
| **29** | ATPB sf |  |  |  | 46056 |  | 5.02 | 1 | 52 | 46 |
| **30** | C1QBP | Complement component 1 Q subcomponent-binding protein, mitochondrial | Q07021 | 31362 | 31046 | 4.74 | 4.33 | 1 | 80 | 26 |
| **31** | CAH1 | Carbonic anhydrase 1 | P00915 | 28870 | 28916 | 6.59 | 6.88 | 1 | 17 | 65 |
| **32** | CALR | Calreticulin | P27797 | 48142 | 63075 | 4.29 | 4.24 | 1 | 29 | 35 |
| **33** | CATD | Cathepsin D | P07339 | 44552 | 29416 | 6.10 | 5.33 | 1 | 39 | 41 |
| **34** | CATD sf |  |  |  | 27804 |  | 6.12 | 1 | 59 | 26 |
| **35** | CAZA1 | F-actin-capping protein subunit alpha-1 | P52907 | 32923 | 34077 | 5.45 | 5.48 | 1 | 22 | 31 |
| **36** | CH10 | 10 kDa heat shock protein, mitochondrial | P61604 | 10932 | 11507 | 8.89 | 7.79 | 1 | 40 | 65 |

| ***** | **Abbreviated Name** | **Protein Name** | **AC Number** | **Theor MW** | **Exp MW** | **Theor pI** | **Exp pI** | **ID methods** | **% Masses Matched** | **Sequence coverage (%) N-terminal residues** |
| --- | --- | --- | --- | --- | --- | --- | --- | --- | --- | --- |
| **37** | CH60 a | 60 kDa heat shock protein, mitochondrial | P10809 | 61055 | 59768 | 5.70 | 4.90 | 1 | 22 | 32 |
| **38** | CH60 b |  |  |  | 59297 |  | 5.06 | 1,2 | 28 | 26, res. 33-38 |
| **39** | CH60 c |  |  |  | 59297 |  | 5.14 | 1 | 78 | 39 |
| **40** | CH60 d |  |  |  | 59500 |  | 5.22 | 2 | - | res 33-38 |
| **41** | CH60 e |  |  |  | 59500 |  | 5.30 | 1,2 | 78 | res 33-38 |
| **42** | CH60 sf |  |  |  | 54818 |  | 5.28 | 1 | 38 | 16 |
| **43** | CLIC1 | Chloride intracellular channel protein 1 | O00299 | 26923 | 29488 | 5.09 | 5.00 | 1 | 48 | 43 |
| **44** | COF1 a | Cofilin-1 | P23528 | 18502 | 26054 | 8.22 | 6.42 | 1 | 45 | 61 |
| **45** | COF1 b |  |  |  | 26669 |  | 6.87 | 1 | 100 | 52 |
| **46** | COF1 c |  |  |  | 26669 |  | 6.81 | 1 | 53 | 42 |
| **47** | COPE | Coatomer subunit epsilon | O14579 | 34482 | 29416 | 4.98 | 5.08 | 1 | 17 | 41 |
| **48** | COX5A | Cytochrome c oxidase polypeptide Va | P20674 | 16762 | 14198 | 6.30 | 4.91 | 1 | 17 | 52 |
| **49** | CYTB sf | Cystatin-B | P04080 | 11140 | 14364 | 6.96 | 6.84 | 1 | 13 | 44 |
| **50** | DDAH2 | N(G),N(G)-dimethylarginine dimethylaminohydrolase 2 | O95865 | 29644 | 28845 | 5.66 | 5.66 | 1 | 27 | 35 |
| **51** | ECHM | Enoyl-CoA hydratase, mitochondrial | P30084 | 31387 | 26734 | 8.34 | 6.00 | 1 | 22 | 39 |
| **52** | EF1A1 | Elongation factor 1-alpha 1 | P68104 | 50141 | 48147 | 9.10 | 8.36 | 1 | 21 | 21 |
| **53** | EF1B | Elongation factor 1-beta | P24534 | 24764 | 29272 | 4.50 | 4.48 | 1 | 50 | 29 |
| **54** | EIF3I | Eukaryotic translation initiation factor 3 subunit I | Q13347 | 36502 | 37121 | 5.38 | 5.47 | 1 | 7 | 48 |
| **55** | ENOA a | Alpha-enolase | P06733 | 47169 | 48477 | 7.01 | 6.25 | 3 | - | - |
| **56** | ENOA b |  |  |  | 48809 |  | 6.42 | 3 | - | - |
| **57** | ENOA c |  |  |  | 49312 |  | 6.64 | 3 | - | - |
| **58** | ENOA d |  |  |  | 49480 |  | 6.88 | 3 | - | - |
| **59** | ENPL | 94 kDa glucose-regulated protein | P14625 | 92469 | 101052 | 4.76 | 4.73 | 1 | 33 | 23 |
| **60** | FABP7 | Fatty acid-binding protein, brain | O15540 | 14889 | 15317 | 5.40 | 5.29 | 1 | 28 | 93 |
| **61** | FSCN1 | Fascin | Q16658 | 54530 | 51550 | 6.84 | 6.61 | 1 | 60 | 29 |
| **62** | FUMH | Fumarate hydratase, mitochondrial | P07954 | 54637 | 46690 | 8.85 | 6.85 | 1 | 14 | 36 |

| ***** | **Abbreviated Name** | **Protein Name** | | **AC Number** | | | | **Theor MW** | | | | | **Exp MW** | | | | **Theor pI** | | | | **Exp pI** | | | | **ID methods** | | | | | **% Masses Matched** | | | **Sequence coverage (%) N-terminal residues** | | | | |
| --- | --- | --- | --- | --- | --- | --- | --- | --- | --- | --- | --- | --- | --- | --- | --- | --- | --- | --- | --- | --- | --- | --- | --- | --- | --- | --- | --- | --- | --- | --- | --- | --- | --- | --- | --- | --- | --- |
| **63** | G3P a | Glyceraldehyde-3-phosphate dehydrogenase | | P04406 | | | | 36053 | | | | | 36215 | | | | 8.57 | | | | 6.94 | | | | 1 | | | | | 73 | | | 26 | | | | |
| **64** | G3P b |  | |  | | | |  | | | | | 35965 | | | |  | | | | 7.15 | | | | 2 | | | | | - | | | res. 2-11 | | | | |
| **65** | G3P c |  | |  | | | |  | | | | | 36053 | | | |  | | | | 7.31 | | | | 2 | | | | | - | | | res. 2-21 | | | | |
| **66** | G3P d |  | |  | | | |  | | | | | 36053 | | | |  | | | | 7.51 | | | | 2 | | | | | - | | | res. 2-11 | | | | |
| **67** | G3P e |  | |  | | | |  | | | | | 35965 | | | |  | | | | 7.71 | | | | 2 | | | | | - | | | res. 2-31 | | | | |
| **68** | GLOD4 | Glyoxalase domain-containing protein 4 | | Q9HC38 | | | | 34793 | | | | | 33910 | | | | 5.40 | | | | 5.38 | | | | 1 | | | | | 17 | | | 47 | | | | |
| **69** | GRP75 | 75 kDa glucose-regulated protein | | P38646 | | | | 73681 | | | | | 72818 | | | | 5.87 | | | | 5.39 | | | | 1 | | | | | 82 | | | 24 | | | | |
| **70** | GRP78 | 78 kDa glucose-regulated protein | | P11021 | | | | 72333 | | | | | 76504 | | | | 5.07 | | | | 4.93 | | | | 1 | | | | | 66 | | | 49 | | | | |
| **71** | GSTO1 | Glutathione transferase omega-1 | | P78417 | | | | 27566 | | | | | 29272 | | | | 6.24 | | | | 6.10 | | | | 1 | | | | | 19 | | | 27 | | | | |
| **72** | GSTP1 a | Glutathione S-transferase P | | P09211 | | | | 23356 | | | | | 26669 | | | | 5.43 | | | | 5.28 | | | | 1 | | | | | 71 | | | 34 | | | | |
| **73** | GSTP1 b |  | |  | | | |  | | | | | 26669 | | | |  | | | | 5.55 | | | | 1 | | | | | 29 | | | 40 | | | | |
| **74** | HSP71 | Heat shock 70 kDa protein 1A/1B | | P08107 | | | | 70052 | | | | | 69622 | | | | 5.47 | | | | 5.46 | | | | 1 | | | | | 38 | | | 38 | | | | |
| **75** | HSP7C a | Heat shock cognate 71 kDa protein | | P11142 | | | | 70898 | | | | | 70566 | | | | 5.37 | | | | 5.24 | | | | 1 | | | | | 22 | | | 21 | | | | |
| **76** | HSP7C b |  | |  | | | |  | | | | | 70566 | | | |  | | | | 5.31 | | | | 1 | | | | | 23 | | | 28 | | | | |
| **77** | HSP7C c |  | |  | | | |  | | | | | 69935 | | | |  | | | | 5.36 | | | | 1 | | | | | 70 | | | 43 | | | | |
| **78** | HSP7C d |  | |  | | | |  | | | | | 73146 | | | |  | | | | 5.46 | | | | 1 | | | | | 38 | | | 38 | | | | |
| **79** | HSPB1 | Heat shock protein beta-1 | | P04792 | | | | 22783 | | | | | 26669 | | | | 5.98 | | | | 5.95 | | | | 1 | | | | | 52 | | | 61 | | | | |
| **80** | IDH3A | Isocitrate dehydrogenase [NAD] subunit alpha, mitochondrial | | P50213 | | | | 39592 | | | | | 37879 | | | | 6.46 | | | | 5.77 | | | | 1 | | | | | 57 | | | 12 | | | | |
| **81** | IDHC | Isocitrate dehydrogenase [NADP] cytoplasmic | | O75874 | | | | 46659 | | | | | 46531 | | | | 6.53 | | | | 6.79 | | | | 1 | | | | | 20 | | | 25 | | | | |
| **82** | IF5A1 | Eukaryotic translation initiation factor 5A | | P63241 | | | | 16832 | | | | | 23048 | | | | 5.07 | | | | 5.12 | | | | 1 | | | | | 38 | | | 67 | | | | |
| **83** | IPYR | Inorganic pyrophosphatase | | Q15181 | | | | 32660 | | | | | 34752 | | | | 5.54 | | | | 5.38 | | | | 1 | | | | | 40 | | | 48 | | | | |
| **84** | K1C10 | Keratin, type I cytoskeletal 10 | | P35527 | | | | 62064 | | | | | 45277 | | | | 5.14 | | | | 5.30 | | | | 1 | | | | | 18 | | | 11 | | | | |
| **85** | K1C9 | Keratin, type I cytoskeletal 9 | | P13645 | | | | 58827 | | | | | 62232 | | | | 5.13 | | | | 6.83 | | | | 1 | | | | | 22 | | | 33 | | | | |
| **86** | KPYM a | Pyruvate kinase isozymes M1/M2 | | P14618 | | | | 57937 | | | | | 80737 | | | | 7.96 | | | | 6.48 | | | | 1 | | | | | 25 | | | 39 | | | | |
| **87** | KPYM b |  | |  | | | |  | | | | | 61953 | | | |  | | | | 6.82 | | | | 1 | | | | | 89 | | | 53 | | | | |
| **88** | LDHA | L-lactate dehydrogenase A chain | | P00338 | | | | 36689 | | | | | 34077 | | | | 8.44 | | | | 7.09 | | | | 1 | | | | | 34 | | | 36 | | | | |
| **89** | LDHB | L-lactate dehydrogenase B chain | | P07195 | | | | 36638 | | | | | 35094 | | | | 5.71 | | | | 5.69 | | | | 1 | | | | | 23 | | | 49 | | | | |
|  |  |  | |  | | | |  | | | | |  | | | |  | | | |  | | | |  | | | | |  | | |  | | | | |
| ***** | **Abbreviated Name** | **Protein Name** | | | | **AC Number** | | | **Theor MW** | | | **Exp MW** | | | | **Theor pI** | | | | **Exp pI** | | | | **ID methods** | | | | **% Masses Matched** | | | | **Sequence coverage (%) N-terminal residues** | | | |  |  |
| **90** | LEG1 | Galectin-1 | | | | P09382 | | | 14716 | | | 13709 | | | | 5.30 | | | | 5.06 | | | | 1 | | | | 32 | | | | 61 | | | |  |  |
| **91** | LGUL | Lactoylglutathione lyase | | | | Q04760 | | | 65331 | | | 26669 | | | | 5.12 | | | | 4.95 | | | | 1 | | | | 24 | | | | 31 | | | |  |  |
| **92** | MIF | Macrophage migration inhibitory factor | | | | P14174 | | | 12476 | | | 12270 | | | | 7.73 | | | | 6.83 | | | | 1 | | | | 80 | | | | 21 | | | |  |  |
| **93** | MIF sf |  | | | |  | | |  | | | 12270 | | | |  | | | | 6.85 | | | | 1 | | | | 20 | | | | 23 | | | |  |  |
| **94** | MYL6 | Myosin light polypeptide 6 | | | | P60660 | | | 16930 | | | 18355 | | | | 4.56 | | | | 4.53 | | | | 1 | | | | 18 | | | | 50 | | | |  |  |
| **95** | NDKA | Nucleoside diphosphate kinase A | | | | P15531 | | | 17149 | | | 26669 | | | | 5.81 | | | | 5.82 | | | | 1 | | | | 22 | | | | 52 | | | |  |  |
| **96** | NDKB | Nucleoside diphosphate kinase B | | | | P22392 | | | 17298 | | | 25902 | | | | 8.52 | | | | 7.29 | | | | 1 | | | | 58 | | | | 56 | | | |  |  |
| **97** | NPM | Nucleophosmin | | | | P06748 | | | 32575 | | | 26669 | | | | 4.64 | | | | 4.47 | | | | 1 | | | | 60 | | | | 23 | | | |  |  |
| **98** | NTF2 | Nuclear transport factor 2 | | | | P61970 | | | 14478 | | | 11307 | | | | 5.10 | | | | 4.92 | | | | 1 | | | | 37 | | | | 53 | | | |  |  |
| **99** | PDIA1 | Protein disulfide-isomerase | | | | P07237 | | | 57116 | | | 56722 | | | | 4.76 | | | | 4.67 | | | | 1 | | | | 33 | | | | 9 | | | |  |  |
| **100** | PDIA3 a | Protein disulfide-isomerase A3 | | | | P30101 | | | 56782 | | | 55006 | | | | 5.98 | | | | 5.53 | | | | 1 | | | | 57 | | | | 21 | | | |  |  |
| **101** | PDIA3 b |  | | | |  | | |  | | | 54818 | | | |  | | | | 5.73 | | | | 1 | | | | 57 | | | | 27 | | | |  |  |
| **102** | PDIA3 c |  | | | |  | | |  | | | 54818 | | | |  | | | | 5.84 | | | | 1 | | | | 21 | | | | 43 | | | |  |  |
| **103** | PGAM1 | Phosphoglycerate mutase 1 | | | | P18669 | | | 28804 | | | 27940 | | | | 6.67 | | | | 6.78 | | | | 1 | | | | 48 | | | | 58 | | | |  |  |
| **104** | PGK 1 a | Phosphoglycerate kinase 1 | | | | P00558 | | | 44615 | | | 44510 | | | | 8.30 | | | | 6.83 | | | | 1 | | | | 26 | | | | 17 | | | |  |  |
| **105** | PGK 1 b |  | | | |  | | |  | | | 44359 | | | |  | | | | 6.91 | | | | 1 | | | | 43 | | | | 28 | | | |  |  |
| **106** | PGK 1 c |  | | | |  | | |  | | | 42432 | | | |  | | | | 7.19 | | | | 1 | | | | 67 | | | | 13 | | | |  |  |
| **107** | PHB | Prohibitin | | | | P35232 | | | 29804 | | | 27736 | | | | 5.57 | | | | 5.51 | | | | 1 | | | | 56 | | | | 58 | | | |  |  |
| **108** | PNPH | Purine nucleoside phosphorylase | | | | P00491 | | | 32118 | | | 29561 | | | | 6.45 | | | | 6.45 | | | | 1 | | | | 24 | | | | 66 | | | |  |  |
| **109** | PNPO | Pyridoxine-5'-phosphate oxidase | | | | Q9NVS9 | | | 29988 | | | 29488 | | | | 6.61 | | | | 6.05 | | | | 1 | | | | 22 | | | | 28 | | | |  |  |
| **110** | PPIA a | Peptidyl-prolyl cis-trans isomerase A | | | | P62937 | | | 18012 | | | 23318 | | | | 7.68 | | | | 6.88 | | | | 1 | | | | 73 | | | | 65 | | | |  |  |
| **111** | PPIA b |  | | | |  | | |  | | | 23183 | | | |  | | | | 6.86 | | | | 1 | | | | 73 | | | | 65 | | | |  |  |
| **112** | PPIA c |  | | | |  | | |  | | | 22780 | | | |  | | | | 6.83 | | | | 1 | | | | 33 | | | | 51 | | | |  |  |
| **113** | PPIB | Peptidyl-prolyl cis-trans isomerase B | | | | P23284 | | | 23743 | | | 26669 | | | | 9.42 | | | | 8.42 | | | | 1 | | | | 85 | | | | 49 | | | |  |  |
| **114** | PRDX1 a | Peroxiredoxin-1 | | | | Q06830 | | | 22110 | | | 26669 | | | | 8.27 | | | | 6.83 | | | | 1 | | | | 63 | | | | 45 | | | |  |  |
| **115** | PRDX1 b |  | | | |  | | |  | | | 26669 | | | |  | | | | 6.96 | | | | 1 | | | | 44 | | | | 18 | | | |  |  |
| **116** | PRDX6 a | Peroxiredoxin 6 | | | | P30041 | | | 25035 | | | 26669 | | | | 6.00 | | | | 5.65 | | | | 1 | | | | 53 | | | | 34 | | | |  |  |
| **117** | PRDX6 b |  | | | |  | | |  | | | 26669 | | | |  | | | | 6.33 | | | | 1 | | | | 17 | | | | 36 | | | |  |  |
| **118** | PROF1 a | Profilin-1 | | | | P07737 | | | 15054 | | | 15317 | | | | 8.44 | | | | 6.86 | | | | 1 | | | | 71 | | | | 40 | | | |  |  |
| **119** | PROF1 c |  | | | |  | | |  | | | 15679 | | | |  | | | | 6.89 | | | | 1 | | | | 72 | | | | 52 | | | |  |  |
| **120** | PRS8 | 26S protease regulatory subunit 8 | | | | P62195 | | | 45626 | | | 46531 | | | | 7.11 | | | | 6.87 | | | | 1 | | | | 62 | | | | 15 | | | |  |  |
| **121** | PSA4 | Proteasome subunit alpha type-4 | | | | P25789 | | | 29484 | | | 28916 | | | | 7.58 | | | | 6.84 | | | | 1 | | | | 16 | | | | 48 | | | |  |  |
| ***** | **Abbreviated Name** | **Protein Name** | | | **AC Number** | | | **Theor MW** | | | **Exp MW** | | | | **Theor pI** | | | | **Exp pI** | | | | **ID methods** | | | | **% Masses Matched** | | | | **Sequence coverage (%) N-terminal residues** | | |  | | |  |
| **122** | PSA5 | Proteasome subunit alpha type-5 | | | P28066 | | | 26411 | | | 26669 | | | | 4.74 | | | | 4.66 | | | | 2 | | | | - | | | | res. 4-13 | | |  | | |  |
| **123** | PSA6 | Proteasome subunit alpha type-6 | | | P60900 | | | 27393 | | | 26932 | | | | 6.34 | | | | 6.37 | | | | 1 | | | | 28 | | | | 35 | | |  | | |  |
| **124** | RABP2 | Cellular retinoic acid-binding protein 2 | | | P29373 | | | 15693 | | | 15771 | | | | 5.38 | | | | 5.56 | | | | 2 | | | | - | | | | res. 1-10 | | |  | | |  |
| **125** | RL40 | Ubiquitin-60S ribosomal protein L40 | | | P62987 | | | 14728 | | | 8594 | | | | 9.87 | | | | 6.89 | | | | 1 | | | | 17 | | | | 47 | | |  | | |  |
| **126** | RUVB1 | RuvB-like 1 | | | Q9Y265 | | | 50228 | | | 52978 | | | | 6.02 | | | | 6.41 | | | | 1 | | | | 47 | | | | 25 | | |  | | |  |
| **127** | S10A2 | Protein S100-A2 | | | P29034 | | | 11117 | | | 10002 | | | | 4.68 | | | | 4.60 | | | | 1 | | | | 89 | | | | 38 | | |  | | |  |
| **128** | S10A6 a | Protein S100-A6 | | | P06703 | | | 10180 | | | 8494 | | | | 5.32 | | | | 4.97 | | | | 1 | | | | 20 | | | | 52 | | |  | | |  |
| **129** | S10A6 b |  | | |  | | |  | | | 8644 | | | |  | | | | 5.12 | | | | 1 | | | | 80 | | | | 40 | | |  | | |  |
| **130** | S10AB | Protein S100-A11 | | | P31949 | | | 11740 | | | 10666 | | | | 6.56 | | | | 6.00 | | | | 1 | | | | 25 | | | | 57 | | |  | | |  |
| **131** | SAHH | Adenosylhomocysteinase | | | P23526 | | | 47716 | | | 46056 | | | | 5.92 | | | | 6.12 | | | | 1 | | | | 14 | | | | 30 | | |  | | |  |
| **132** | SERPH | Serpin H1 | | | P50454 | | | 46441 | | | 45277 | | | | 8.75 | | | | 8.12 | | | | 1 | | | | 13 | | | | 39 | | |  | | |  |
| **133** | SH3L1 | SH3 domain-binding glutamic acid-rich-like protein | | | O75368 | | | 12774 | | | 13951 | | | | 5.22 | | | | 5.29 | | | | 2 | | | | - | | | | res. 2-11 | | |  | | |  |
| **134** | SH3L3 | SH3 domain-binding glutamic acid-rich-like protein 3 | | | Q9H299 | | | 10438 | | | 10420 | | | | 4.82 | | | | 4.84 | | | | 1 | | | | 11 | | | | 41 | | |  | | |  |
| **135** | SODM | Superoxide dismutase [Mn], mitochondrial | | | P04179 | | | 24722 | | | 26669 | | | | 8.35 | | | | 6.87 | | | | 2 | | | | - | | | | res. 25-34 | | |  | | |  |
| **136** | TBA1A a | Tubulin alpha-1 chain | | | Q71U36 | | | 50136 | | | 54074 | | | | 4.94 | | | | 4.95 | | | | 1 | | | | 57 | | | | 51 | | |  | | |  |
| **137** | TBA1A b |  | | |  | | |  | | | 54445 | | | |  | | | | 5.00 | | | | 1 | | | | 76 | | | | 52 | | |  | | |  |
| **138** | TBA1A c |  | | |  | | |  | | | 54259 | | | |  | | | | 5.05 | | | | 1 | | | | 57 | | | | 30 | | |  | | |  |
| **139** | TBB5 a | Tubulin beta-5 chain | | | P07437 | | | 49671 | | | 51727 | | | | 4.78 | | | | 4.77 | | | | 1 | | | | 52 | | | | 58 | | |  | | |  |
| **140** | TBB5 b |  | | |  | | |  | | | 51727 | | | |  | | | | 4.82 | | | | 1 | | | | 84 | | | | 60 | | |  | | |  |
| **141** | TCPA | T-complex protein 1 subunit alpha | | | P17987 | | | 60344 | | | 57699 | | | | 5.80 | | | | 5.92 | | | | 1 | | | | 55 | | | | 13 | | |  | | |  |
| **142** | TCPB a | T-complex protein 1 subunit beta | | | P78371 | | | 57488 | | | 54259 | | | | 6.01 | | | | 6.10 | | | | 1 | | | | 27 | | | | 19 | | |  | | |  |
| **143** | TCPB b |  | | |  | | |  | | | 54445 | | | |  | | | | 6.20 | | | | 1 | | | | 16 | | | | 49 | | |  | | |  |
| **144** | TCTP | Translationally controlled tumor protein | | | P13693 | | | 19595 | | | 26669 | | | | 4.84 | | | | 4.85 | | | | 1 | | | | 24 | | | | 33 | | |  | | |  |
| **145** | TERA | Transitional endoplasmic reticulum ATPase | | | P55072 | | | 89322 | | | 112546 | | | | 5.14 | | | | 5.28 | | | | 1 | | | | 31 | | | | 17 | | |  | | |  |
| **146** | TERA sf |  | | |  | | |  | | | 96183 | | | |  | | | | 5.12 | | | | 1 | | | | 67 | | | | 40 | | |  | | |  |
| **147** | THIO a | Thioredoxin | | | P10599 | | | 11738 | | | 12782 | | | | 4.82 | | | | 4.84 | | | | 1 | | | | 100 | | | | 72 | | |  | | |  |
| **148** | THIO b | Thioredoxin | | | P10599 | | | 11738 | | | 12707 | | | | 4.82 | | | | 4.95 | | | | 1 | | | | 41 | | | | 88 | | |  | | |  |
| **149** | TPIS a | Triosephosphate isomerase | | | P60174 | | | 30791 | | | 26669 | | | | 5.65 | | | | 6.18 | | | | 1 | | | | 22 | | | | 38 | | |  | | |  |
| **150** | TPIS b |  | | |  | | |  | | | 26669 | | | |  | | | | 6.24 | | | | 1 | | | | 77 | | | | 38 | | |  | | |  |
| **151** | TPIS c |  | | |  | | |  | | | 26669 | | | |  | | | | 6.50 | | | | 1 | | | | 15 | | | | 28 | | |  | | |  |
| **152** | TPIS d |  | | |  | | |  | | | 26669 | | | |  | | | | 6.68 | | | | 2 | | | | - | | | | res.3-11 | | |  | | |  |
| **153** | TPIS e |  | | |  | | |  | | | 26669 | | | |  | | | | 6.81 | | | | 1 | | | | 100 | | | | 8 | | |  | | |  |
| **154** | TPIS sf |  | | |  | | |  | | | 26669 | | | |  | | | | 4.89 | | | | 1 | | | | 66 | | | | 55 | | |  | | |  |
| ***** | **Abbreviated Name** | | **Protein Name** | | | | **AC Number** | | | **Theor MW** | | | | **Exp MW** | | | | **Theor pI** | | | | **Exp pI** | | | | **ID methods** | | | **% Masses Matched** | | | | **Sequence coverage (%) N-terminal residues** | |  | |  |
| **155** | TPM1 | | Tropomyosin alpha-1 chain | | | | P09493 | | | 32709 | | | | 32209 | | | | 4.69 | | | | 4.55 | | | | 1 | | | 21 | | | | 36 | |  | |  |
| **156** | TPM2 | | Tropomyosin beta chain | | | | P07951 | | | 32850 | | | | 36378 | | | | 4.66 | | | | 4.61 | | | | 1 | | | 26 | | | | 20 | |  | |  |
| **157** | TPM4 | | Tropomyosin alpha-4 chain | | | | P67936 | | | 28521 | | | | 30294 | | | | 4.67 | | | | 4.63 | | | | 1 | | | 28 | | | | 38 | |  | |  |
| **158** | UBE2N | | Ubiquitin-conjugating enzyme E2 N | | | | P61088 | | | 17138 | | | | 18789 | | | | 6.13 | | | | 5.91 | | | | 1 | | | 89 | | | | 51 | |  | |  |
| **159** | UCHL1 | | Ubiquitin carboxyl-terminal hydrolase isozyme L1 | | | | P09936 | | | 24824 | | | | 26669 | | | | 5.33 | | | | 5.42 | | | | 1 | | | 71 | | | | 67 | |  | |  |
| **160** | VDAC1 | | Voltage-dependent anion-selective channel protein 1 | | | | P21796 | | | 30773 | | | | 32766 | | | | 8.62 | | | | 7.67 | | | | 1 | | | 76 | | | | 73 | |  | |  |
| **161** | VIME | | Vimentin | | | | P08670 | | | 53652 | | | | 60037 | | | | 5.05 | | | | 4.76 | | | | 1 | | | 15 | | | | 19 | |  | |  |
| **162** | VIME fr | |  | | | |  | | |  | | | | 46531 | | | |  | | | | 4.55 | | | | 1 | | | 51 | | | | 43 | |  | |  |
| **163** | VINC a | | Vinculin | | | | P18206 | | | 123799 | | | | 119845 | | | | 5.50 | | | | 5.99 | | | | 1 | | | 16 | | | | 26 | |  | |  |
| **164** | VINC b | |  | | | |  | | |  | | | | 120384 | | | |  | | | | 6.05 | | | | 1 | | | 25 | | | | 30 | |  | |  |

**Additional Table 1.** - **Catalogue of the protein spots identified in the 2-D-IPG prototype map of f-LSCs.**

Table reports the following information: Protein names, accession numbers (AC) and abbreviated names correspond to the nomenclature used in the Swiss-Prot database. The experimental values of pI and MW for every isoelectric spot were calculated with ImageMaster 2D Platinum system; the theoretical values represent the predicted MW and pI for each identified protein according to Swiss-Prot and TrEMBL database. Identification methods: 1, MALDI-TOF; 2, N-terminal sequencing by automated Edman degradation and 3, Western Blotting.
