# Supplementary material for: Donor age and long-term culture do not negatively influence the stem potential of limbal fibroblast-like stem cells
Source: Stem Cell Res Ther. 2016 Jun 13;7:83. doi: 10.1186/s13287-016-0342-z (PMC4906894; doi:10.1186/s13287-016-0342-z)
Supplement: Additional file 1: — Table S2 Catalogue of the unvaried and differential expressed proteins in f-LSCs samples. (ZIP 110 kb) [file 13287_2016_342_MOESM1_ESM.zip › Additional file 1.doc]

| **INVARIED PROTEINS** | |  |  |  | **DIFFERENTIAL PROTEINS** | |  |  |
| --- | --- | --- | --- | --- | --- | --- | --- | --- |
| *Landmark* | ***AC*** | *Landmark* | ***AC*** |  | *Landmark* | ***AC*** | *Landmark* | ***AC*** |
| ACBP | P07108 | S10A6 a | P06703 |  | ACTB 6 | P60709 | SODM | P04179 |
| ACTB 4 | P60709 | S10AB | P31949 |  | ANXA2 a | P07355 | TBA1 c | Q71U36 |
| AL1A1 | P00352 | SH3L3 | Q9H299 |  | ANXA4 b | P09525 | TCPA | P17987 |
| ALDOA a | P04075 | TBA1 a | Q71U36 |  | ANXA5 a | P48036 | TCPB 21 | P78371 |
| ANXA1 | P04083 | TBB5 a | P07437 |  | ARPC5 | O15511 | TERA 26 | P55072 |
| ANXA2 b | P07355 | TCTP | P13693 |  | ATPB sf | P06576 | THIO a | P10599 |
| ATPB | P06576 | TERA a | P55072 |  | CAH1 | P00915 | TPIS a | P60174 |
| C1QBP | Q07021 | THIO b | P10599 |  | CATD a | P07339 | UCHL1 | P09936 |
| CALR | P27797 | TPM1 | P09493 |  | CH60 43 | P10809 | VDAC1 | P21796 |
| CAZA1 | P52907 | TPM2 7 | P07951 |  | COF1 a | P23528 | VIME 40 | P08670 |
| CH10 | P61604 | TPM4 22 | P67936 |  | CYTB sf | P04080 | VINC a | P18206 |
| CH60 b | P10809 | UBE2N | P61088 |  | ECHM | P30084 |  |  |
| CLIC1 | O00299 |  |  |  | FABP7 | O15540 |  |  |
| COF1 c | P23528 |  |  |  | FUMH | P07954 |  |  |
| COPE | O14579 |  |  |  | G3P a | P04406 |  |  |
| COX5A | P20674 |  |  |  | GLOD4 | Q9HC38 |  |  |
| DDAH2 | O95865 |  |  |  | GSTO1 b | P78417 |  |  |
| EF1A1 | P68104 |  |  |  | GSTP1 a | P09211 |  |  |
| EF1B | P24534 |  |  |  | IDH3A | P50213 |  |  |
| EIF3I | Q13347 |  |  |  | IDHC | O75874 |  |  |
| ENOA a | P06733 |  |  |  | IF5A1 | P63241 |  |  |
| ENPL | P14625 |  |  |  | K1C10 28 | P13645 |  |  |
| FSCN1 | Q16658 |  |  |  | LDHA | P00338 |  |  |
| G3P c | P04406 |  |  |  | LDHB | P07195 |  |  |
| GRP75 | P38646 |  |  |  | LGUL | Q04760 |  |  |
| GRP78 | P11021 |  |  |  | MIF | P14174 |  |  |
| GSTP1 b | P09211 |  |  |  | MYL6 | P60660 |  |  |
| HSP71 | P08107 |  |  |  | NDKB | P22392 |  |  |
| HSP7C a | P11142 |  |  |  | NTF2 | P61970 |  |  |
| HSPB1 a | P04792 |  |  |  | PDIA1 | P07237 |  |  |
| IPYR | Q15181 |  |  |  | PDIA3 37 | P30101 |  |  |
| K1C9 | P35527 |  |  |  | PGK 1 a | P00558 |  |  |
| KPYM a | P14618 |  |  |  | PHB | P35232 |  |  |
| LEG1 | P09382 |  |  |  | PNPH | P00491 |  |  |
| NDKA | P15531 |  |  |  | PNPO | Q9NVS9 |  |  |
| NPM | P06748 |  |  |  | PPIA b | P62937 |  |  |
| PGAM1 b | P18669 |  |  |  | PPIB | P23284 |  |  |
| PRDX1 a | Q06830 |  |  |  | PRDX1 b | Q06830 |  |  |
| PRDX6 a | P30041 |  |  |  | PRDX6 b | P30041 |  |  |
| PROF1 a | P07737 |  |  |  | PRS8 | P62195 |  |  |
| PSA4 | P25789 |  |  |  | RABP2 | P29373 |  |  |
| PSA5 | P28066 |  |  |  | RL40 | P62987 |  |  |
| PSA6 | P60900 |  |  |  | SAHH | P23526 |  |  |
| RUVB1 | Q9Y265 |  |  |  | SERPH | P50454 |  |  |
| S10A2 | P29034 |  |  |  | SH3L1 | Q9H299 |  |  |
